# Dietary Macronutrient Composition and Protein Concentration for Weight Loss Maintenance

**DOI:** 10.1002/oby.24370

**Published:** 2025-08-07

**Authors:** Hanyue Zhang, Aikaterina Vasileiou, Dominique Searle, Sofus C. Larsen, Alistair M. Senior, Faidon Magkos, Leigh C. Ward, Graham Horgan, Inês Santos, António L. Palmeira, Stephen J. Simpson, David Raubenheimer, R. James Stubbs, Berit L. Heitmann

**Affiliations:** ^1^ Research Unit for Diet and Health The Parker Institute, Bispebjerg and Frederiksberg Hospital Frederiksberg Denmark; ^2^ Department of Food Science University of Copenhagen Copenhagen Denmark; ^3^ Center for Clinical Research and Prevention Bispebjerg and Frederiksberg Hospital Frederiksberg Denmark; ^4^ Section for General Practice, Department of Public Health University of Copenhagen Copenhagen Denmark; ^5^ Charles Perkins Centre University of Sydney Sydney New South Wales Australia; ^6^ School of Life and Environmental Sciences University of Sydney Sydney New South Wales Australia; ^7^ Sydney Precision Data Science Centre University of Sydney Sydney New South Wales Australia; ^8^ Department of Nutrition, Exercise and Sports University of Copenhagen Copenhagen Denmark; ^9^ School of Chemistry and Molecular Biosciences The University of Queensland Brisbane Queensland Australia; ^10^ Biomathematics and Statistics Scotland, BioSS Aberdeen UK; ^11^ Laboratório de Nutrição, Faculdade de Medicina, Centro Académico de Medicina de Lisboa Universidade de Lisboa Lisbon Portugal; ^12^ Instituto de Saúde Ambiental (ISAMB), Faculdade de Medicina Universidade de Lisboa Lisbon Portugal; ^13^ CIDEFES, Universidade Lusófona & CIFI2D, Universidade do Porto Portugal; ^14^ School of Psychology, Faculty of Medicine and Health University of Leeds Leeds UK

**Keywords:** cohort study, dietary protein, obesity prevention, weight loss maintenance

## Abstract

**Objective:**

To examine the association between dietary macronutrient composition and 12‐month weight loss maintenance (WLM) in adults who achieved initial weight loss (≥ 5%).

**Methods:**

This prospective cohort analysis used 12‐month follow‐up data from the Navigating to a Healthy Weight trial. Macronutrient composition (%) was assessed using a 4‐day, 24‐h dietary recall. Food sources were categorized as discretionary foods, lean meat, vegetables, fruit, grains, and dairy. Primary outcomes included 12‐month changes in body weight, fat mass index (FMI), waist‐to‐height ratio (WHtR), and hip‐to‐height ratio (HHtR). A nutritional geometry approach was used to examine individual and interactive associations of macronutrient intake, visualizing as response surfaces.

**Results:**

Among 1518 participants (69.8% women; mean age 45 ± 12 years), mean macronutrient composition was 20.6% protein, 33.8% fat, and 43.1% carbohydrate. Protein energy percentage was inversely associated with energy intake (β: −0.33; 95% CI: −0.39, −0.27). Response surfaces revealed that lower proportional energy from protein, diluted by high fat and/or carbohydrate, was associated with higher total energy intake and greater 12‐month increases in body weight, WHtR, and HHtR, but not FMI. Consumption of discretionary food, not other food sources, increased energy intake by reducing proportional energy from protein.

**Conclusions:**

Maintaining dietary proportional energy from protein, particularly by limiting discretionary food consumption, was associated with reduced energy intake and improved WLM.

**Study Importance**

- What is already known?
  ○ What represents an optimal macronutrient composition for long‐term weight loss maintenance (WLM) remains an open question.
  ○ Studies have suggested that protein intake is tightly controlled in humans, which drives excess food and energy intake when dietary protein is diluted by other energy‐yielding macronutrients, termed “protein leverage.”
  ○ However, the role of dietary macronutrient composition and protein leverage in long‐term WLM remained unclear.
- What does this study add?
  ○ In the prospective analysis of 1518 adults with initial weight loss (≥ 5%), nutritional geometry revealed that a lower percentage of energy from protein, diluted by high fat and/or carbohydrate, was associated with higher total food and energy intake and greater 12‐month increases in body weight, waist‐height ratio, and hip‐height ratio.
  ○ Additionally, high consumption of discretionary foods was associated with increased energy intake by lowering the percentage of energy from protein in the diet.
- How might these results change the direction of research or the focus of clinical practice?
  ○ Avoiding dietary protein dilution, particularly through low consumption of discretionary foods, may decrease food and energy intake and benefit long‐term WLM.

## 1 Introduction

Obesity is a growing public health problem [1] and a leading cause of noncommunicable diseases [2]. While numerous interventions have proven effective in achieving initial weight loss [3, 4], maintaining long‐term weight loss (WLM) remains challenging [5, 6]. It is widely agreed that dietary macronutrient composition may influence energy intake and weight gain [7, 8]. However, the optimal macronutrient balance for long‐term WLM remains unclear. Although much focus has been on dietary fat and carbohydrate [9, 10], there is growing recognition of the role of dietary protein in regulating energy intake and weight gain [11, 12]. Emerging evidence suggested that humans [13, 14, 15, 16, 17], like other species [18, 19], regulate protein intake more tightly than other macronutrients. When dietary protein concentration (percentage of energy) is diluted by other energy‐yielding macronutrients, total food and energy intake increase disproportionally, a phenomenon termed “protein leverage” [11, 12]. Evidence for protein leverage has been widely observed in the adult general population [13, 14, 15, 16, 17] and children [20, 21]. However, the specific role of protein leverage and macronutrient composition in long‐term WLM remains unclear.

The consumption of certain foods, particularly those that dilute dietary protein, may significantly interact with the protein leverage mechanism, potentially leading to excessive energy intake and weight gain [12]. Data from the NHANES 2009–2010 dietary recall showed that ultraprocessed food intake was associated with dietary protein dilution, along with increased consumption of fat, carbohydrate, and total energy, while absolute protein intake remained stable [16]. Similarly, a comprehensive analysis of the Australian National Nutrition and Physical Activity Survey (NNPAS) [17], using a geometric framework [22], revealed that discretionary foods were low in protein and had intermediate fat‐to‐carbohydrate ratios. High energy intake was associated with high discretionary food consumption but not with other food groups [17]. Despite these findings, there are few studies examining specific food groups as the ecological contributors to dietary protein dilution and their impact on long‐term WLM.

Using data from a large WLM trial across three European centers, “Navigating to a Healthy Weight” (NoHoW), we aimed to: (i) investigate the role of dietary macronutrient composition and protein leverage in long‐term WLM and (ii) identify specific food groups that are associated with dietary protein dilution, driving excessive energy intake, for the application of more precise recommendations regarding food sources.

## 2 Methods

### 2.1 Study Population

The study is a secondary analysis of data from the NoHoW trial [23], a three‐center (UK, Denmark, and Portugal), 2 × 2 factorial, single‐blind randomized controlled trial to test the efficacy of a digital toolkit for WLM. The trial included participants [23] who were 18 years or older, had achieved a clinically significant weight loss (≥ 5%) within the 12 months prior to inclusion, and had a pre‐weight loss body mass index (BMI) of ≥ 25 kg/m^2^. A total of 1627 participants were recruited between 2017 and 2018, with the primary follow‐up visit, including weight and health biomarker measurements, occurring 12 months after enrollment. The NoHoW trial was conducted in accordance with the Helsinki Declaration. Ethical approval was obtained from the local institutional ethics committees at the Universities of Leeds (17‐0082), Lisbon (17/2016), and the Capital Region of Denmark (H16030495) [23].

From the original 1627 participants, we excluded those with missing baseline dietary data (*n* = 58) and those reporting implausible energy intake, outside the ranges of 500–3500 kcal/day (2.09–14.65 MJ/day) for women and 800–4000 kcal/day (3.35–16.74 MJ/day) for men [24] (*n* = 51). These exclusions left us with 1518 men and women, representing 93% of the total NoHoW trial. Baseline anthropometric data were available for 1513 participants for body weight, 1496 for body composition, and 1510 and 1511 for waist and hip circumference, respectively. At the 12‐month follow‐up, the corresponding numbers were 1200, 1177, 1194, and 1196 (Figure S1).

### 2.2 Exposures

Dietary information was collected at baseline and the 12‐month follow‐up using a 4‐day, 24‐h dietary recall via the online platform INTAKE24, which included at least one weekend day [23, 25]. A country‐specific food composition database was used to estimate daily macronutrient intakes [23]. The primary exposures were the proportional energy intakes from protein, fat, and carbohydrate at baseline, analyzed as continuous variables (percentage of energy).

Food groups were classified according to the Australian Dietary Guidelines [26] into five food groups and discretionary foods. The five food groups include (1) lean meats, poultry, fish, eggs, tofu, nuts, and seeds; (2) grains and cereals; (3) vegetables; (4) fruit; and (5) milk, yogurt, and cheese. Discretionary foods were defined as energy‐dense, high in saturated fat, added sugars, salt, or alcohol, and low in fiber [26]. Examples include cakes, biscuits, confectionery, deep‐fried fast foods, processed meats, and sweetened beverages (see online Supporting Information Text S1).

### 2.3 Outcomes

The primary outcomes were the 12‐month changes in body weight (kg), fat mass index (FMI), waist‐height ratio (WHtR), and hip‐height ratio (HHtR), calculated as the difference between baseline and follow‐up values. At baseline and after the 12 months of follow‐up, body weight and height were measured to the nearest 0.1 kg and 0.1 cm using the Seca 704 s scale, stadiometer, and electronic scale [23]. Body composition was measured using bioelectrical impedance analysis using the ImpediMed SFB7 device. Fat mass was determined using the Moissl BMI modification of the mixture theory equations [27, 28]. Waist and hip circumferences were measured twice to the nearest 0.5 cm, and if the measures differed by more than 1 cm, a third measurement was taken [23]. FMI was calculated as fat mass in kilograms divided by height in meters squared. WHtR and HHtR were calculated as the measures of waist and hip circumference (cm), respectively, divided by height (cm). Changes in primary outcomes (follow‐up values minus baseline values), that is, 12‐month WLM, were included in analyses as continuous variables, including changes in body weight (kg), FMI (kg/m^2^), WHtR, and HHtR.

### 2.4 Statistical Analysis

Participants' characteristics across quintiles of energy proportion from protein were presented as mean (standard deviation, SD) for continuous variables and frequency (percentage, %) for categorical variables.

We employed mixture models from Lawson and Willden [29], using energy proportions from protein, fat, and carbohydrate as the primary exposures, to estimate the role of dietary macronutrient composition and interactions in total food intake (dry weight), energy intake, and 12‐month WLM. The associations were visualized on right‐angle mixture triangles (RMT), a three‐dimensional nutritional geometry, as response surfaces [30], with color gradients indicating outcome values—red for higher and blue for lower values. Three models were used: Model 1 (null model, no dietary association), Model 2 (additive linear association, or “partition substitution model” in nutritional epidemiology), and Model 3 (non‐linear quadratic association). Model fit was assessed using the Akaike information criterion (AIC), with lower AIC scores indicating a better fit. All models were adjusted for covariates selected based on previous research [31, 32, 33], including age, sex, fiber intake, initial weight loss, baseline fat‐free mass, energy expenditure (estimated using Fitbit Charge 2) [23], baseline BMI (< 25, 25–< 30, or ≥ 30 kg/m^2^), education (low, medium, high, or other), alcohol consumption frequency (every day, 5–6 times a week, 3–4 times a week, twice a week, once a week, or less than once a week), trial arm (control, self‐regulation and motivation, stress and emotion regulation, or stress and emotion regulation plus self‐regulation and motivation), and country (UK, Denmark, or Portugal). Analyses were conducted on complete cases. Additionally, we illustrated the macronutrient composition of each food group by plotting the proportional energy from protein, fat, and carbohydrate as scatter points on the RMT [17, 22].

To estimate leverage strength for energy intake, we used power function regression using the proportion of energy from protein, fat, and carbohydrate as the respective exposures. The regression equation is: $E=P\times p^{L}$, where *E* represents total energy intake, *P* is a constant, *p* is the proportion of energy from dietary protein, fat, or carbohydrate, and *L* is leverage strength (with *P* and *L* being estimated variables) [34, 35]. Models were adjusted for the covariates mentioned earlier. The Benjamini–Hochberg procedure [36] was applied to control the false discovery rate for multiple comparisons.

We used generalized additive models [37] to examine the association between the proportion of food groups in the diet (discretionary foods and the five food groups) and total energy intake, with results shown as smooth trend lines. Four models were fitted: Model a was unadjusted. Model (b) adjusted for covariates. Model (c) further adjusted for saturated fat, sodium, and sugar intake, and Model d additionally included the percentage of energy from protein. Continuous covariates were *z*‐transformed and modeled as smooth terms, while categorical covariates were included as factor parametric terms. To control for the false discovery rate due to multiple comparisons across the different models, we applied the Benjamini–Hochberg procedure [36].

### 2.5 Sensitivity Analyses

To test the robustness of our findings, we conducted sensitivity analyses. First, since approximately 20% of participants had dietary data from only 1 to 3 days instead of the full 4‐day baseline period, we repeated the primary analyses after excluding these participants. Second, for missing covariates (about 15% in the multivariable model), we compared the characteristics of participants retained in the complete‐case analysis with those who were excluded and used multiple imputations with chained equations [38], generating 10 imputed datasets and reanalyzed the data accordingly (online Supporting Information Text S2). Third, we considered the potential for reporting bias [39]. A previous study on the NoHoW trial [40] indicated that total energy intake estimated via INTAKE24 was lower than energy expenditure measured by Fitbit Charge 2, suggesting underreporting. We had previously reported that underreporting is more pronounced for fat‐ and carbohydrate‐rich foods than for protein‐rich foods, especially among individuals with obesity [39, 41, 42]. To evaluate the potential influence of systematic underreporting, we conducted a sensitivity analysis adjusting for underreported energy intake in participants with obesity under three different assumptions (details in online Supporting Information Text S3). All statistical analyses were conducted using R (version 4.1.2). A 2‐tailed test with *p* < 0.05 was considered statistically significant.

## 3 Results

The analysis included 1518 participants, of whom 1060 (69.8%) were women, with a mean (SD) age of 45 (12) years. Participants in the highest quintile of percentage of energy from protein (≥ 24.9%) were older (45 compared to 43 years), had greater initial weight loss (13.3 compared to 10.5 kg), consumed less fiber (8.4 compared to 9.9 g/day), and were more likely to be from Denmark and Portugal than the UK (38.9% and 32.6% compared to 28.6%) than those in the lowest quintile (< 15.9%). Additionally, those in the lowest protein quintile showed the highest regains in body weight, body weight regains in relation to initial weight loss, FMI, WHtR, and HHtR (Table 1).

**TABLE 1:** Characteristics of study participants by quintiles of energy proportion from protein.

	All participants (*n* = 1518)	Quintile groups of energy proportion from protein (%)				
		< 15.9 (*n* = 304)	15.9–< 18.6 (*n* = 303)	18.6–< 21.3 (*n* = 304)	21.3–< 24.9 (*n* = 303)	≥ 24.9 (*n* = 301)
Age, mean (SD), years	45 (11.9)	43 (11.4)	45 (11.7)	46 (12.1)	45 (12.0)	45 (12.0)
Fiber intake, mean (SD), g	9.1 (8.6)	9.9 (7.7)	9.3 (8.5)	9.6 (9.1)	8.3 (8.1)	8.4 (9.5)
Fat‐free mass, mean (SD), kg	52.1 (10.1)	51.3 (9.4)	52.9 (10.2)	51.1 (9.7)	52.2 (10.3)	53.0 (10.5)
Energy expenditure, mean (SD), MJ	11.6 (2.6)	11.3 (2.3)	11.6 (2.6)	11.3 (2.6)	11.7 (2.6)	11.9 (2.7)
Initial weight loss, mean (SD), kg	11.6 (6.5)	10.5 (5.6)	10.9 (5.6)	11.3 (7.0)	12.3 (6.8)	13.3 (7.1)
Sex, no. (%)						
Male	458 (30.2)	81 (26.6)	104 (34.3)	84 (27.6)	101 (33.3)	87 (28.9)
Female	1060 (69.8)	223 (73.4)	199 (65.7)	220 (72.4)	202 (66.7)	214 (71.1)
BMI at baseline, no. (%)^a^						
< 25 kg/m^2^	278 (18.4)	57 (18.8)	60 (19.9)	59 (19.5)	57 (18.9)	44 (14.7)
25–< 30 kg/m^2^	633 (41.8)	140 (46.1)	126 (41.9)	133 (43.9)	121 (40.1)	112 (37.3)
≥ 30 kg/m^2^	602 (39.8)	107 (35.2)	115 (38.2)	111 (36.6)	124 (41.1)	144 (48.0)
Country, no. (%)						
UK (Leeds)	535 (35.2)	140 (46.1)	103 (34.0)	117 (38.5)	89 (29.4)	86 (28.6)
Denmark (Copenhagen)	500 (32.9)	98 (32.2)	97 (32.0)	88 (29.0)	98 (32.3)	117 (38.9)
Portugal (Lisbon)	483 (31.8)	66 (21.7)	103 (34.0)	99 (32.6)	116 (38.3)	98 (32.6)
Education, no. (%)						
Low	136 (9.2)	22 (7.5)	21 (7.1)	30 (10.3)	32 (10.8)	31 (10.4)
Medium	314 (21.2)	53 (18.0)	71 (24.0)	63 (21.6)	69 (23.3)	58 (19.5)
High	1030 (69.6)	220 (74.6)	204 (68.9)	199 (68.2)	195 (65.9)	209 (70.1)
Frequency of alcohol consumption, no. (%)						
Every day	34 (2.3)	7 (2.4)	5 (1.7)	6 (2.0)	12 (4.0)	4 (1.3)
5–6 times a week	50 (3.4)	11 (3.7)	14 (4.7)	14 (4.8)	6 (2.0)	5 (1.7)
3–4 times a week	151 (10.1)	31 (10.5)	35 (11.7)	27 (9.2)	32 (10.7)	26 (8.7)
Twice a week	225 (15.1)	47 (15.9)	47 (15.7)	45 (15.3)	38 (12.8)	46 (15.3)
Once a week	167 (11.2)	31 (10.5)	35 (11.7)	38 (12.9)	33 (11.1)	30 (10.0)
< Once a week	863 (57.9)	168 (57.0)	163 (54.5)	165 (55.9)	177 (59.4)	189 (63.0)
Trial arm, no. (%)						
Control	375 (24.7)	71 (23.4)	77 (25.4)	80 (26.3)	71 (23.4)	75 (24.9)
Self‐regulation and motivation	386 (25.4)	79 (26.0)	71 (23.4)	70 (23.0)	83 (27.4)	81 (26.9)
Stress and emotion regulation	375 (24.7)	72 (23.7)	84 (27.7)	80 (26.3)	70 (23.1)	69 (22.9)
Stress and emotion regulation + self‐regulation and motivation	382 (25.2)	82 (27.0)	71 (23.4)	74 (24.3)	79 (26.1)	76 (25.3)
Total energy intake, mean (SD), MJ	7.4 (2.6)	8.1 (2.5)	7.9 (2.7)	7.5 (2.6)	7.2 (2.5)	6.3 (2.3)
Total fat intake, mean (SD), MJ	2.5 (1.2)	2.8 (1.3)	2.8 (1.3)	2.5 (1.1)	2.4 (1.1)	2.1 (1.1)
Total carbohydrate intake, mean (SD), MJ	3.2 (1.3)	3.8 (1.3)	3.5 (1.3)	3.3 (1.2)	2.9 (1.1)	2.3 (1.0)
Changes in body weight, mean (SD), kg	0.4 (6.1)	1.3 (5.1)	0.1 (5.4)	0.2 (5.9)	0.6 (6.8)	‐0.2 (6.9)
% changes in body weight/initial weight loss, mean (SD)	3.1 (61.6)	10.3 (56.6)	−0.3 (56.3)	3.2 (63.3)	2.6 (69.5)	−0.2 (61.1)
Changes in FMI, mean (SD), kg/m^2^	−0.1 (2.2)	0.1 (2.1)	−0.2 (1.9)	0.0 (2.2)	−0.2 (2.4)	−0.1 (2.4)
Changes in WHtR, mean (SD)	0.1 (3.9)	0.5 (3.7)	−0.4 (3.8)	0.3 (3.7)	0.2 (4.0)	−0.3 (4.3)
Changes in HHtR, mean (SD)	0.1 (3.4)	0.0 (3.1)	0.0 (3.2)	0.4 (3.3)	0.4 (3.8)	−0.3 (3.6)

Abbreviations: FMI, fat mass index; HHtR, hip‐height ratio; WHtR, waist height ratio.

^a^ Calculated as weight in kilograms divided by height in meters squared.

At baseline, the mean (SD) energy proportions were 20.6% (5.8) for protein, 33.8% (8.8) for fat, and 43.1% (10.8) for carbohydrate. These values were comparable to those at the 12‐month follow‐up: 20.9% (6.2) for protein, 35.2% (9.8) for fat, and 43.9% (10.5) for carbohydrate (Table S1). The distribution of macronutrient intake at baseline is illustrated in Figure S2a, with protein showing the narrowest range compared to fat and carbohydrate (interquartile ranges of 7.2%, 10.7%, and 14.0%, respectively). Figure S2b plots protein versus non‐protein intake, showing a near‐vertical line, consistent with protein leverage, indicating less variability in protein intake compared to fat and carbohydrate.

### 3.1 Dietary Macronutrient Composition, Energy Intake, and 12‐Month WLM

**FIGURE 1:**
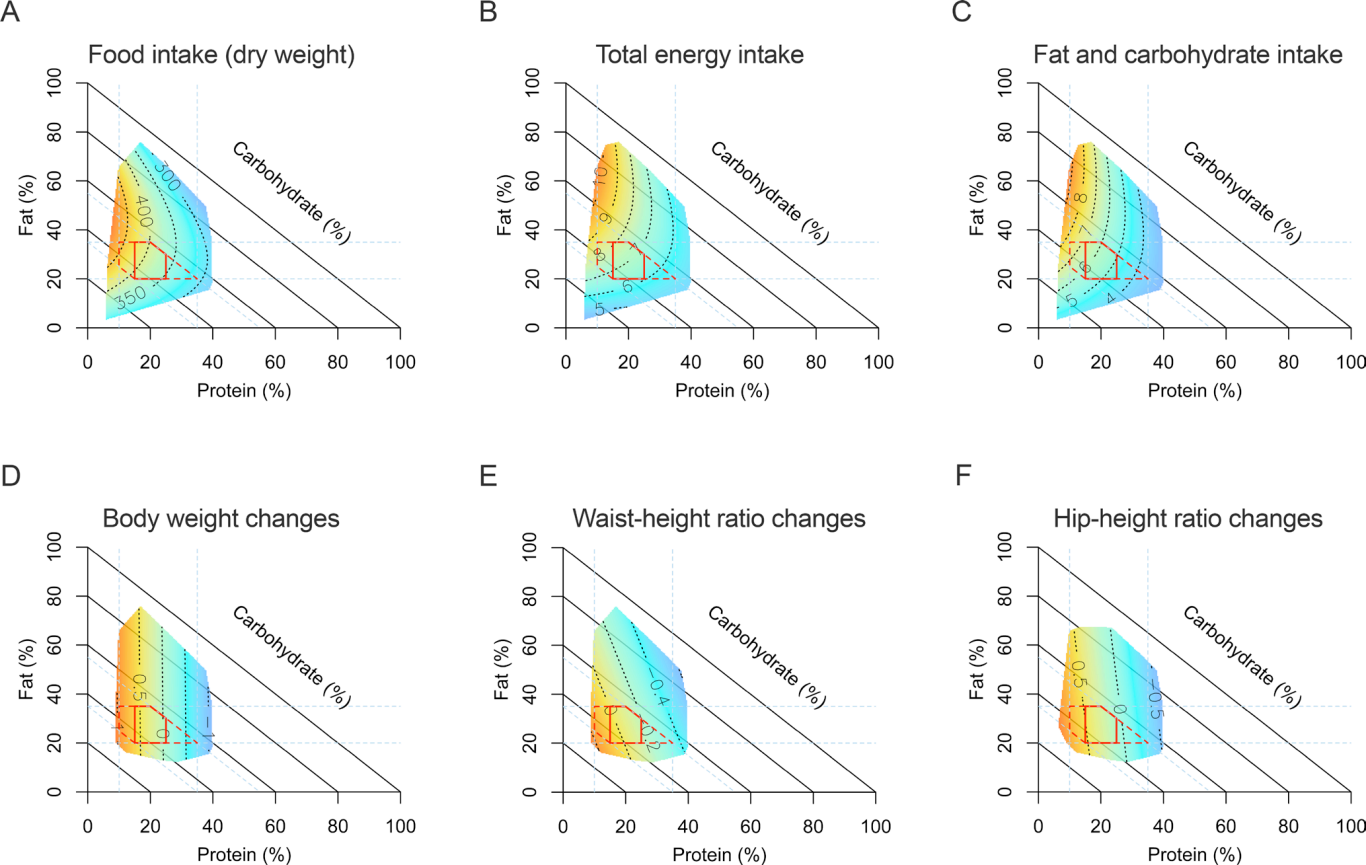
Dietary macronutrient composition in relation to total food and energy intake and 12‐month weight loss maintenance. Right‐angled mixture triangle (RMT) plots show the association between macronutrient composition (% of energy) and predicted (A) total food intake (dry weight, g), (B) total energy intake (MJ), (C) fat and carbohydrate intake (MJ), and 12‐month changes in (D) body weight (kg) and (E) waist‐ and (F) hip‐height ratio. In the RMT, proportional energy from protein (%*P*), fat (%*F*), and carbohydrate (%*C*) sum to 100%. While %*P* and %*F* increase along their respective axes, %*C* increases across the diagonal lines with decreasing distance from the origin. On the plots, red represents the highest, while blue represents the lowest value of the outcomes. Mixture models estimated colored surfaces. Models were adjusted for age, sex, initial weight loss, fiber intake, energy expenditure, BMI at baseline, fat‐free mass at baseline, education, frequency of alcohol consumption, trial arm, and country. Statistics are given in Tables S2a and S2b. For reference, the red solid polygon represents the Acceptable Macronutrient Distribution Range (AMDR) for Australia and New Zealand (%*p* = 15–25, %*F* = 20–35, %*C* = 45–65), and the red dotted polygon in the background shows the AMDR for the United States (%*p* = 10–35, %*F* = 20–35, %*C* = 45–65). [Color figure can be viewed at wileyonlinelibrary.com]

The associations between dietary macronutrient composition and intakes of total food (dry weight), total energy, and fat and carbohydrate are illustrated in Figure 1A–C. The quadratic model (Model 3) was preferred for all three outcomes, as shown in Tables S2a and S2b. The response surfaces align with the protein axis in the RMTs, indicating the percentage of energy from protein as the primary predictor. A lower percentage of energy from protein was associated with higher intakes of total food (dry weight), total energy, and fat and carbohydrate. The leverage strength, indicated by the L coefficient, was strongest for protein, with values of (−0.30; 95% CI: −0.36, −0.24) for total food, (−0.33; 95% CI: −0.39, −0.27) for total energy, and (−0.56; 95% CI: −0.62, −0.50) for fat and carbohydrate intakes (Table S3 and Figure S3).

**FIGURE 2:**
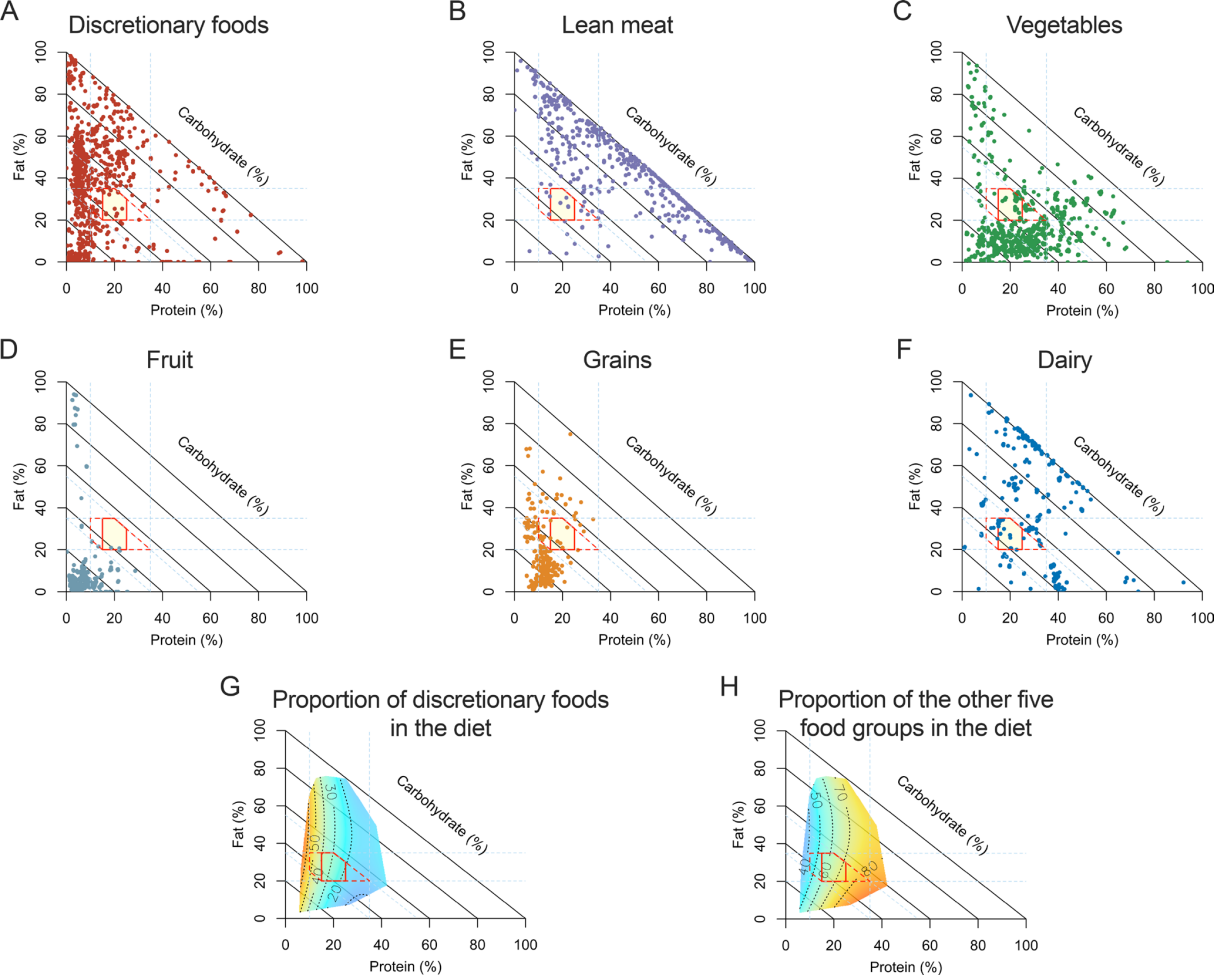
Macronutrient composition of discretionary foods and the five food groups. (A–F) Right‐angled mixture triangle (RMT) plots show the macronutrient composition of each food group. In the RMT, proportional energy from protein (%*P*), fat (%*F*), and carbohydrate (%*C*) sum to 100%. While %*P* and %*F* increase along their respective axes, %*C* increases across the diagonal lines with decreasing distance from the origin. The data points in RMT indicate the proportion of energy from protein, fat, and carbohydrate of foods in each food group. Description values are given in Table S4. (G–H) RMT plots show the association of macronutrient composition (% of energy) with the predicted energy proportion from discretionary foods and the five food groups. On the plots, red represents the highest, while blue represents the lowest value of the outcomes. Mixture models estimated colored surfaces. Models were adjusted for age, sex, initial weight loss, fiber intake, energy expenditure, BMI at baseline, fat‐free mass at baseline, education, frequency of alcohol consumption, trial arm, and country. Statistics are given in Tables S5a and S5b. For reference, the red solid polygon represents the Acceptable Macronutrient Distribution Range (AMDR) for Australia and New Zealand (%*p* = 15–25, %*F* = 20–35, %*C* = 45–65), and the red dotted polygon in the background shows the AMDR for the United States (%*p* = 10–35, %*F* = 20–35, %*C* = 45–65). [Color figure can be viewed at wileyonlinelibrary.com]

The fully adjusted associations between dietary macronutrient composition and 12‐month WLM are depicted in Figure 1D–F. The AIC favored the additive linear model (Model 2) for predicting changes in body weight, WHtR, and HHtR, while the null model for FMI, which indicated no dietary association (Tables S2a and S2b). The response surfaces in Figure 2D–F align with the protein axis in the RMTs, indicating that a lower percentage of energy from protein was associated with increases in body weight, WHtR, and HHtR after 12 months.

### 3.2 Macronutrient Composition and Protein Leverage in Food Groups

The macronutrient composition of various food groups is shown in Figure 2. Discretionary foods clustered in areas with a low percentage of energy from protein (median 4.4%) and either a high percentage of energy from fat (35.4%) or carbohydrate (43.3%) (Figure 2A and Table S4). In contrast, the five food groups, such as lean meats, dairy, and vegetables, showed a higher percentage of energy from protein, ranging from 18.5% to 39.6% (Figure 2B–F and Table S4). Figure 2G,H demonstrate the associations between dietary macronutrient composition and the intake of discretionary foods and the five food groups. The response surfaces align with the protein axis in the RMTs. A lower percentage of energy from protein was associated with higher consumption of discretionary foods, while a higher percentage of energy from protein was associated with greater intake of the five food groups (Tables S5a and S5b).

As consumption of discretionary foods increased across quintiles (from < 15.2 to ≥ 43.8% energy), the percentage of energy from protein decreased (from a median of 24.1% to 15.9%). Meanwhile, non‐protein energy (from fat and carbohydrates) and total energy intake increased, with medians rising from 4.8 and 6.6 MJ to 6.2 and 7.8 MJ, respectively. However, absolute protein intake remained relatively stable at around 1.4 MJ (Figure S4 and Table S6). Moreover, we observed significant direct associations between discretionary food consumption and total energy intake across unadjusted and multivariable‐adjusted models (*p* < 0.001, model a–c). However, after adjusting for the percentage of energy from protein, this association was no longer significant (*p* = 0.09, model d). Similarly, the inverse associations between the consumption of the five food groups and total energy intake, which were significant in models (a–c) (*p* < 0.001), also disappeared after adjusting for the percentage of energy from protein (*p* = 0.11, model d) (Figure S5 and Table S7).

### 3.3 Sensitivity Analyses

First, excluding participants with fewer than 4 days of dietary data (the remaining 1194 participants) yielded results consistent with the full sample (Figure S6). Second, participants retained in the complete‐case analysis were comparable to those excluded in most characteristics, including age, fiber intake, initial weight loss, baseline fat‐free mass, baseline BMI, education, alcohol consumption frequency, and trial arm. However, differences were observed in sex and country (Table S8). Associations derived from multiple imputation models were similar to those from models with missing data (Figure S7). Third, after adjusting for potential underreporting among participants with obesity, the RMT plots for total food (dry weight), total energy, and fat and carbohydrate intake showed patterns consistent with the main results (Figure S8). For 12‐month WLM, although different patterns in the RMT plots with different underreporting assumptions for fat and carbohydrate intake, the greatest regains in body weight, WHtR, and HHtR consistently occurred where dietary protein concentration was lowest.

## 4 Discussion

In this prospective analysis of 1518 adults with initial weight loss, we found evidence supporting the protein leverage hypothesis in 12‐month WLM. The protein leverage hypothesis proposes that the dilution of protein in modern food supplies by fat and carbohydrate‐rich highly processed foods has resulted in increased energy intake through protein leverage, increasing weight gain and obesity risk [12]. In our study, protein intake showed less variability than fat and carbohydrate intake, suggesting tight regulation of protein intake. Using nutritional geometry, we observed that a lower percentage of energy from protein, diluted by high fat and/or carbohydrate, was associated with higher total food and energy intake and greater 12‐month increases in body weight, WHtR, and HHtR. These findings are consistent with the protein leverage hypothesis, suggesting that lower protein concentration in the diet may promote excess energy consumption and impair long‐term weight maintenance. Moreover, high consumption of discretionary foods was associated with increased energy intake by reducing the protein concentration in the diet.

Our findings are consistent with previous randomized controlled trials suggesting that high‐protein diets effectively support WLM. For example, the Diet, Obesity, and Gene's study [43] showed that a modest increase in dietary protein and a slight reduction in glycemic index led to optimal weight maintenance over 26 weeks, with participants on high‐protein diets regaining about 1 kg less than those on low‐protein diets. Other studies have similarly found that diets providing 20%–35% of energy from protein are associated with less weight regain compared to diets with 10%–20% of energy from protein [44]. Our results for dietary protein on total food (dry weight) and energy intake are also consistent with previous research on protein leverage, including studies in the general population [13, 14, 15, 16, 17], among children [20, 21], and an analysis of data from 38 published experimental trials [12].

In adults with initial weight loss, we found that high consumption of discretionary foods increased energy intake by lowering dietary protein concentration. These findings align with previous studies among the general population using NHANES 2009–2010 dietary data in the United States [16] and data from the NNPAS in Australia [17]. Our findings suggest that reducing the intake of discretionary foods to prevent protein dilution may benefit long‐term WLM. However, it's important to recognize that eating behavior is influenced by a complex interplay of factors, including the neurobiology of eating, energy homeostasis, hedonics, and the dual aspects of reflective and reactive behavior [45]. Therefore, other mechanisms may work independently or synergistically with protein leverage to influence food intake and WLM.

This study has several strengths. First, it was based on data from a large WLM trial, enhancing the statistical power and reliability of the findings. The prospective design minimizes recall bias, as dietary information was collected before assessing WLM outcomes. Additionally, comprehensive data on lifestyle and health‐related factors allowed us to examine the robustness of our findings across strata of characteristics. Our results remained consistent after adjusting for multiple lifestyle factors and were validated through sensitivity analyses. Lastly, using a multidimensional approach, nutritional geometry, enabled us to examine both individual and interactive associations of the three macronutrients on outcomes.

The study has some potential limitations. First, despite adjusting for many potential confounders, residual or unmeasured confounding cannot be entirely ruled out. Second, although most participants provided 4‐day dietary data, a small number provided only 1–3 days, which could lead to misclassification. However, excluding those with fewer than 4 days of data yielded similar results in the remaining 1225 participants. Furthermore, dietary intake assessed at baseline was comparable to that at the 12‐month follow‐up, suggesting the consistency of dietary intake over 12 months. Third, as with other dietary studies, reporting bias may be a concern [40]. Nevertheless, this bias tends to be less pronounced for protein intake than for carbohydrate or fat [39, 41, 42], and it did not affect the conclusions on protein leverage [35]. Additionally, our sensitivity analyses, which explored various assumptions about systematic underreporting among participants with obesity, yielded results consistent with the main findings. Last, our results are based on a specific group of participants who received a 12‐month digital intervention. Although we adjusted for the trial arm in all analyses, caution is needed when generalizing these findings to the broader population.

## 5 Conclusion

We found that lower dietary protein concentration was associated with higher total food and energy intake and subsequent increases in body weight, WHtR, and HHtR over 12 months. High consumption of discretionary foods contributed to increased energy intake, primarily by reducing dietary protein concentration. These findings suggest that reducing the intake of discretionary foods to prevent protein dilution may benefit long‐term WLM.

## Author Contributions

Hanyue Zhang and Berit L. Heitmann had full access to all of the data in the study and take responsibility for the integrity of the data and the accuracy of the data analysis. Concept and design: Hanyue Zhang, Stephen J. Simpson, David Raubenheimer, Berit L. Heitmann. Methodology (development or design of methodology; creation of models): Hanyue Zhang, Alistair M. Senior, Stephen J. Simpson, David Raubenheimer. Acquisition, analysis, or interpretation of data: Hanyue Zhang, Stephen J. Simpson, David Raubenheimer, Berit L. Heitmann. Drafting of the manuscript: Hanyue Zhang, Aikaterina Vasileiou, Dominique Searle. Critical revision of the manuscript for important intellectual content: Hanyue Zhang, Aikaterina Vasileiou, Dominique Searle, Sofus C. Larsen, Alistair M. Senior, Faidon Magkos, Leigh C. Ward, Graham Horgan, Inês Santos, António L. Palmeira, Stephen J. Simpson, David Raubenheimer, R. James Stubbs, Berit L. Heitmann. Statistical analysis: Hanyue Zhang. Administrative, technical, or material support: Sofus C. Larsen, Leigh C. Ward, Graham Horgan. Supervision: Stephen J. Simpson, David Raubenheimer, Berit L. Heitmann.

## Conflicts of Interest

The authors declare no conflicts of interest.

## Supporting information


**Table S1.** Macronutrient intake at baseline and 12‐month follow‐up.
**Table S2a.** AIC three mixture‐models for associations of macronutrient composition with food intake, energy intake, and 12‐month weight loss maintenance.
**Table S2b.** Coefficients for associations of macronutrient composition with food intake, energy intake, and 12‐month weight loss maintenance.
**Table S3.** The exponent (L) from power regression testing protein prioritization.
**Table S4.** Macronutrient composition (% of energy) in food groups.
**Table S5a.** AIC three mixture models for associations between macronutrient composition and energy intake from discretionary foods and the five food groups.
**Table S5b.** Coefficients for associations between macronutrient composition and energy intake from discretionary foods and the five food groups.
**Table S6.** Macronutrient composition and energy intake across quintiles of discretionary food consumption.
**Table S7.** Generalized additive model outputs for food group consumption and total energy intake.
**Table S8.** Comparison between participants retained in the complete‐case analysis and the excluded participants.
**Figure S1.** Flowchart of the study population.
**Figure S2.** Variability of macronutrient intake.
**Figure S3.** Association of proportional energy from each macronutrient with total food and energy intake.
**Figure S4.** Protein and non‐protein energy intake by quintile groups of discretionary food consumption.
**Figure S5.** Association of proportion of discretionary foods and the five food groups with total energy intake.
**Figure S6.** Association of dietary macronutrient composition with food and energy intake and 12‐month weight loss maintenance after excluding participants with dietary information < 4 days.
**Figure S7.** Association of dietary macronutrient composition with food and energy intake and 12‐month weight loss maintenance after multiple imputations for missing covariates.
**Figure S8.** Association of dietary macronutrient composition with food and energy intake and 12‐month weight loss maintenance after adjusting underreporting.

## Data Availability

The data that support the findings of this study are available on request from the corresponding author. The data are not publicly available due to privacy or ethical restrictions.
