# Supplementary material for: Dietary Macronutrient Composition and Protein Concentration for Weight Loss Maintenance
Source: Obesity (Silver Spring). 2025 Aug 7;33(10):1995–2004. doi: 10.1002/oby.24370 (PMC12477111; doi:10.1002/oby.24370)
Supplement: Supplementary file 1 — Table S1. Macronutrient intake at baseline and 12‐month follow‐up. Table S2a. AIC three mixture‐models for associations of macronutrient composition with food intake, energy intake, and 12‐month weight loss maintenance. Table S2b. Coefficients for associations of macronutrient composition with food intake, energy intake, and 12‐month weight loss maintenance. Table S3. The exponent (L) from power regression testing protein prioritization. Table S4. Macronutrient composition (% of energy) in food groups. Table S5a. AIC three mixture models for associations between macronutrient composition and energy intake from discretionary foods and the five food groups. Table S5b. Coefficients for associations between macronutrient composition and energy intake from discretionary foods and the five food groups. Table S6. Macronutrient composition and energy intake across quintiles of discretionary food consumption. Table S7. Generalized additive model outputs for food group consumption and total energy intake. Table S8. Comparison between participants retained in the complete‐case analysis and the excluded participants. Figure S1. Flowchart of the study population. Figure S2. Variability of macronutrient intake. Figure S3. Association of proportional energy from each macronutrient with total food and energy intake. Figure S4. Protein and non‐protein energy intake by quintile groups of discretionary food consumption. Figure S5. Association of proportion of discretionary foods and the five food groups with total energy intake. Figure S6. Association of dietary macronutrient composition with food and energy intake and 12‐month weight loss maintenance after excluding participants with dietary information < 4 days. Figure S7. Association of dietary macronutrient composition with food and energy intake and 12‐month weight loss maintenance after multiple imputations for missing covariates. Figure S8. Association of dietary macronutrient composition with food and energy intake and 12‐mont [file OBY-33-1995-s001.docx]

**SUPPLEMENTAL MATERIALS**

**Dietary Macronutrient Composition and Protein Concentration for Weight Loss Maintenance**

Hanyue Zhang, Aikaterina Vasileiou, Dominique Searle, Sofus C Larsen, Alistair M. Senior, Faidon Magkos, Leigh Ward, Graham Horgan, Inês Santos, António L Palmeira, Stephen J. Simpson, David Raubenheimer, R James Stubbs, Berit L Heitmann^*^.

^1^ Research Unit for Dietary Studies, The Parker Institute, Bispebjerg and Frederiksberg Hospital, Frederiksberg, Denmark.

^2^ Department of Food Science, University of Copenhagen, Copenhagen, Denmark.

^3^ Center for Clinical Research and Prevention, Bispebjerg and Frederiksberg Hospital, Frederiksberg, Denmark.

^4^ Charles Perkins Centre, University of Sydney, Sydney, New South Wales, Australia.

^5^ School of Life and Environmental Sciences, University of Sydney, Sydney, New South Wales, Australia.

^6^ Sydney Precision Data Science Centre, University of Sydney, Sydney, New South Wales, Australia.

^7^ Department of Nutrition, Exercise and Sports, University of Copenhagen, Copenhagen, Denmark.

^8^ School of Chemistry and Molecular Biosciences, The University of Queensland, Brisbane, Queensland, Australia.

^9^ Biomathematics and Statistics Scotland, BioSS, Aberdeen, United Kingdom.

^10^ Laboratório de Nutrição, Faculdade de Medicina, Centro Académico de Medicina de Lisboa, Universidade de Lisboa, Lisbon, Portugal.

^11^ Instituto de Saúde Ambiental (ISAMB), Faculdade de Medicina, Universidade de Lisboa, Lisbon, Portugal.

^12^ CIDEFES, Universidade Lusófona & CIFI2D, Universidade do Porto, Portugal.

^13^ School of Psychology, Faculty of Medicine and Health, University of Leeds, Leeds, United Kingdom.

^14^ Section for General Practice, Department of Public Health, University of Copenhagen, Copenhagen, Denmark.

**^*^CORRESPONDING AUTHOR:** Berit L. Heitmann. Address: Nordre Fasanvej 57, Vej 8, Entrance 11, 2000 Frederiksberg, Denmark. E-mail: [Berit.Lilienthal.Heitmann@regionh.dk](mailto:Berit.Lilienthal.Heitmann@regionh.dk).

**TABLE OF CONTENTS**

[SUPPLEMENTAL METHODS 4](#_Toc195520485)

[Text S1. Foods in the category of discretionary foods are classified according to the Australian Dietary Guidelines ^1^. 4](#_Toc195520486)

[Text S2. Approach to Missing Data: Use of multiple imputation for missing data in covariates 5](#_Toc195520487)

[Text S3. Sensitivity analysis: Assessing the influence of potential systematic underreporting. 6](#_Toc195520488)

[SUPPLEMENTAL TABLES 7](#_Toc195520489)

[Table S1. Macronutrient intake at baseline and 12-month follow-up. 7](#_Toc195520490)

[Table S2a. AIC three mixture-models for associations of macronutrient composition with food intake, energy intake, and 12-month weight loss maintenance. 8](#_Toc195520491)

[Table S2b. Coefficients for associations of macronutrient composition with food intake, energy intake, and 12-month weight loss maintenance. 8](#_Toc195520492)

[Table S3. The exponent (L) from power regression testing protein prioritization. 10](#_Toc195520493)

[Table S4. Macronutrient composition (% of energy) in food groups. 11](#_Toc195520494)

[Table S5a. AIC three mixture models for associations between macronutrient composition and energy intake from discretionary foods and the five food groups. 12](#_Toc195520495)

[Table S5b. Coefficients for associations between macronutrient composition and energy intake from discretionary foods and the five food groups. 12](#_Toc195520496)

[Table S6. Macronutrient composition and energy intake across quintiles of discretionary food consumption. 13](#_Toc195520497)

[Table S7. Generalized additive model outputs for food group consumption and total energy intake. 14](#_Toc195520498)

[Table S8. Comparison between participants retained in the complete-case analysis and the excluded participants. 15](#_Toc195520499)

[SUPPLEMENTAL FIGURES 16](#_Toc195520500)

[Figure S1. Flowchart of the study population. 16](#_Toc195520501)

[Figure S2. Variability of macronutrient intake. 17](#_Toc195520502)

[Figure S3. Association of proportional energy from each macronutrient with total food and energy intake. 18](#_Toc195520503)

[Figure S4. Protein and non-protein energy intake by quintile groups of discretionary food consumption. 19](#_Toc195520504)

[Figure S5. Association of proportion of discretionary foods and the five food groups with total energy intake. 20](#_Toc195520505)

[Figure S6. Association of dietary macronutrient composition with food and energy intake and 12-month weight loss maintenance after excluding participants with dietary information <4 days. 21](#_Toc195520506)

[Figure S7. Association of dietary macronutrient composition with food and energy intake and 12-month weight loss maintenance after multiple imputations for missing covariates. 22](#_Toc195520507)

[Figure S8. Association of dietary macronutrient composition with food and energy intake and 12-month weight loss maintenance after adjusting underreporting. 24](#_Toc195520508)

[SUPPLEMENTAL REFERENCES 25](#_Toc195520509)

# **SUPPLEMENTAL METHODS**

## **Text S1. Foods in the category of discretionary foods are classified according to the Australian Dietary Guidelines ^1^.**

| **Higher added sugars** | **Higher fat** | **Higher fat and added sugars** | **High alcohol** |
| --- | --- | --- | --- |
| Energy drinks | Bacon, ham | Biscuits | Beer |
| Fruit drinks | Butter, cream, ghee | Cake | Liqueurs |
| Honey | Certain tacos, nachos, enchilada | Chocolate/Bars | Mixed alcoholic drinks |
| Jams, marmalade | Crisps | Dessert custards | Port |
| Some sauces | Dairy blends | Doughnuts | Sherry |
| Sugar | Frankfurts etc | Ice cream | Spirits |
| Sugar confectionary | Meat pies | Iced Buns | Wines |
| Sweetened soft drinks and cordials | Pastry | Muesli bars |  |
| Sweetened waters | Pizza | Puddings |  |
| Syrups | Potato chips | Slices |  |
|  | Quiche | Some confectionaries |  |
|  | Salami/mettwurst | Some sauces/ dressings |  |
|  | Some processed meats | Sweet muffins |  |
|  | Some sauces/dressings | Sweet pastries |  |
|  | Spring roll | Sweet pies and crumbles |  |

## **Text S2. Approach to Missing Data: Use of multiple imputation for missing data in covariates**

The main results presented are from complete case analyses. Approximately 15% of the observations had missing covariate information in the multivariable analyses, with initial weight loss (0.5%, n = 7), fat-free mass at baseline (1.4%, n = 22), energy expenditure (14.3%, n = 217), BMI at baseline (0.3%, n = 5), education (2.1%, n = 32), frequency of alcohol consumption (1.8%, n = 28). As missing data results in the listwise deletion in regression models of observations that have missing values, the missingness may introduce bias, reduce power, and/or affect the representativeness of the results ^2^. In dealing with missing data on covariates, firstly, we presumed the missingness of those covariates was missing at random. We used multiple imputations to address missing data. Multiple imputation with 10 imputed data sets based on a multiple chained equations approach ^3^ was used to fill in the missing values for the covariates above. Secondly, we repeated the main analyses based on the mixture model with right-angled mixture triangles.

There were no notable differences in associations or estimates between the multivariable model results with missing variables and the multiple imputation multivariable model results. Thus, the results of complete case analyses were robust, and these results were not sensitive to missing data in covariates. Furthermore, as we found significant associations from complete case analyses between dietary protein concentration and weight loss maintenance, we considered that using multiple imputations would not add additional information. Results from complete case analyses were therefore presented as our study’s primary results.

## **Text S3. Sensitivity analysis: Assessing the influence of potential systematic underreporting.**

A prior analysis of the OPEN study ^4^ revealed that total energy intake estimated by 24-hour dietary recall was substantially lower than values measured by doubly labeled water, especially among participants with obesity (BMI ≥ 30kg/m^2^), indicating systematic underreporting energy intake. The difference values between the 24-hour dietary recall and doubly labeled water were 2,862 kJ in males and 2,669 kJ in females. Moreover, the underreporting was disproportionate, with the underreporting of fat- and carbohydrate-rich foods being much more substantial than that of protein-rich foods ^4-6^.

Thus, to assess the influence of underreporting energy intake in our study, we conducted a sensitivity analysis by adjusting energy intake using three assumptions among participants with obesity at baseline (BMI ≥ 30kg/m^2^). We assumed:

1) Total energy underreporting was 100% caused by underreporting dietary fat.

Or 2) Total energy underreporting was 100% caused by underreporting dietary carbohydrate.

Or 3) Total energy underreporting was 50% caused by underreporting dietary fat and 50% caused by carbohydrate.

Then, we repeated the main analyses using the adjusted values of proportional energy intake from three macronutrients.

# **SUPPLEMENTAL TABLES**

## **Table S1. Macronutrient intake at baseline and 12-month follow-up.**

|  | **Protein** | **Fat** | **Carbohydrate** |
| --- | --- | --- | --- |
| **At baseline** |  |  |  |
| Mean energy proportion, mean (SD), % | 20.9 (5.9) | 35.5 (9.1) | 43.6 (10.4) |
| Distribution of intake, % |  |  |  |
| Intake quartile 1 | <16.7 | <29.6 | <37.5 |
| Intake quartile 2 | 16.7 – 20.1 | 29.6 – 34.7 | 37.5 – 44.6 |
| Intake quartile 3 | 20.1 – 24.3 | 34.7 – 40.7 | 44.6 – 50.5 |
| Intake quartile 4 | >24.3 | >40.7 | >50.5 |
| IQR ^1^ | 7.6 | 11.1 | 13.0 |
| **12-month follow-up ^2^** |  |  |  |
| Mean energy proportion, mean (SD), % | 20.8 (5.9) | 35.2 (9.7) | 44.0 (10.4) |

^1^ IQRs of macronutrient intake distribution is the difference between the 75th and 25th intake percentile (quartile3 - quartile1). Abbreviation: IQR, Interquartile ranges; SD, standard deviation.

^2^ There was no significant difference in the energy proportion from protein, fat, and carbohydrate between baseline and 12-month follow-up, with p-value = 0.81, 0.40, 0.36, respectively.

## **Table S2a.** **AIC three mixture-models for associations of macronutrient composition with food intake, energy intake, and 12-month weight loss maintenance.**

|  | Model 1  (Null model) | Model 2  (Linear additive model) | Model 3  (Quadratic model) |
| --- | --- | --- | --- |
| Food intake | 13331 | 13233 | **13219** |
| Total energy intake | 5503 | 5342 | **5332** |
| Fat and carbohydrate intake | 5055 | 4702 | **4685** |
| Body weight changes | 6517 | **6512** | 6517 |
| Fat mass index changes | **4203** | 4206 | 4211 |
| Waist-height ratio changes | 5617 | **5613** | 5617 |
| Hip-height ratio changes | 5380 | **5377** | 5378 |

AIC of models that were used to select the best-fitted model for assessing the association of macronutrient composition (% of energy) with food intake, energy intake, and 12-month weight loss maintenance. Model 1: null model (no dietary association); Model 2: linear additive associations (the ‘partition substitution model’ in nutritional epidemiology); and Model 3: quadratic (non-linear) associations. Models with minimal AIC scores are favored. When two models were within two AIC points of one another, the simplest model was selected. AIC Favoured in bold. Abbreviation: AIC, Akaike information criterion.

## **Table S2b. Coefficients for associations of macronutrient composition with food intake, energy intake, and 12-month weight loss maintenance.**

|  | Estimate | SE | t value | p-value |
| --- | --- | --- | --- | --- |
| **Food intake** (dry weight, g) |  |  |  |  |
| *Model 3^1^: R^2^ = 29.6% ^2^, overall p<0.001* | |  |  |  |
| Protein | -190.80 | 374.87 | -0.51 | 0.61 |
| Carbohydrate | -215.05 | 129.01 | -1.67 | 0.10 |
| Fat | -175.95 | 171.47 | -1.03 | 0.31 |
| Protein: Fat | -630.84 | 667.95 | -0.94 | 0.35 |
| Protein: Carbohydrate | -388.68 | 513.23 | -0.76 | 0.45 |
| Carbohydrate: Fat | 891.85 | 215.80 | 4.13 | <0.001 |
| **Total energy intake** (MJ) | |  |  |  |
| *Model 3: R^2^ = 35.4%, overall p<0.001* | |  |  |  |
| Protein | -6.37 | 7.40 | -0.86 | 0.39 |
| Carbohydrate | -8.02 | 2.59 | -3.10 | 0.002 |
| Fat | 1.17 | 3.36 | 0.35 | 0.73 |
| Protein: Fat | -22.60 | 13.12 | -1.72 | 0.09 |
| Protein: Carbohydrate | -1.79 | 10.08 | -0.18 | 0.86 |
| Carbohydrate: Fat | 12.32 | 4.21 | 2.92 | 0.004 |
| **Fat and carbohydrate intake** (MJ) | |  |  |  |
| *Model 3: R^2^ = 41.5%, overall p<0.001* | |  |  |  |
| Protein | 2.03 | 5.69 | 0.36 | 0.72 |
| Carbohydrate | 2.14 | 1.99 | 1.08 | 0.28 |
| Fat | 10.55 | 2.59 | 4.08 | <0.001 |
| Protein: Fat | -29.40 | 10.10 | -2.91 | 0.004 |
| Protein: Carbohydrate | -7.69 | 7.76 | -0.99 | 0.32 |
| Carbohydrate: Fat | 10.13 | 3.24 | 3.12 | 0.002 |
| **Body weight changes** |  |  |  |  |
| *Model 2^3^: R^2^ = 3.6%, overall p<0.001* | |  |  |  |
| Protein | -8.76 | 4.81 | -1.82 | 0.07 |
| Carbohydrate | -1.95 | 4.09 | -0.48 | 0.63 |
| Fat | -1.82 | 4.30 | -0.42 | 0.67 |
| **Waist-height ratio changes** |  |  |  |  |
| *Model 2: R^2^ = 3.6%, overall p<0.001* | |  |  |  |
| Protein | -6.67 | 3.19 | -2.09 | 0.04 |
| Carbohydrate | -4.83 | 2.70 | -1.79 | 0.07 |
| Fat | -4.95 | 2.84 | -1.74 | 0.08 |
| **Hip-height ratio changes** |  |  |  |  |
| *Model 2: R^2^ = 3.5%, overall p<0.001* | |  |  |  |
| Protein | 1.83 | 2.90 | 0.63 | 0.53 |
| Carbohydrate | 5.80 | 2.45 | 2.37 | 0.02 |
| Fat | 5.82 | 2.59 | 2.25 | 0.02 |

^1^ Model 3: quadratic (non-linear) model.

^2^ Adjusted R-squared.

^3^ Model 2: linear additive model.

All models were adjusted for age, sex, initial weight loss, fiber intake, energy expenditure, BMI at baseline, fat-free mass at baseline, education, frequency of alcohol consumption, trial arm, and country. Abbreviation: AIC, Akaike information criterion; Standard Error (SE).

## **Table S3. The exponent (L) from power regression testing protein prioritization.**

|  | **Protein (%)** | **Fat (%)** | **Carbohydrate (%)** |
| --- | --- | --- | --- |
| Food intake (dry weight, g) |  |  |  |
| L ^1^ | -0.30 | 0.07 | 0.15 |
| 95% CI | (-0.36, -0.24) | (0.00, 0.13) | (0.09, 0.21) |
| P value | <0.001 | 0.04 | <0.001 |
| Corrected P value ^2^ | <0.001 | 0.04 | <0.001 |
| Adjusted R-squared | 28.1% | 21.6% | 23.1% |
| Total energy intake (MJ) |  |  |  |
| L | -0.33 | 0.27 | -0.04 |
| 95% CI | (-0.39, -0.27) | (0.21, 0.34) | (-0.1, -0.02) |
| P value | <0.001 | <0.001 | 0.15 |
| Corrected P value | <0.001 | <0.001 | 0.15 |
| Adjusted R-squared | 35.1% | 29.0% | 24.9% |
| Fat and carbohydrate intake (MJ) |  |  |  |
| L | -0.56 | 0.34 | 0.11 |
| 95% CI | (-0.62, -0.50) | (0.28, 0.42) | (0.05, 0.18) |
| P value | <0.001 | <0.001 | 0.001 |
| Corrected P value | <0.001 | <0.001 | 0.001 |
| Adjusted R-squared | 37.3% | 26.1% | 21.0% |

^1^ L indicates strength of leverage for each macronutrient (-1 signifies complete leverage, 0 means no leverage), derived from the log-log regression analysis (power functions).

^2^ P values corrected for 9 comparisons using the Benjamini-Hochberg procedure.

All models were adjusted for age, sex, initial weight loss, fiber intake, energy expenditure, BMI at baseline, fat-free mass at baseline, education, frequency of alcohol consumption, trial arm, and country.

## **Table S4.** **Macronutrient composition (% of energy) in food groups.**

|  | Protein (%) | Fat (%) | Carbohydrate (%) |
| --- | --- | --- | --- |
|  | Median (Q1-Q3) | Median (Q1-Q3) | Median (Q1-Q3) |
| Discretionary foods | 4.4 (0.6-10.5) | 35.4 (0.0-53.9) | 43.3 (4.0-69.5) |
| Lean meat | 39.6 (25.7-64.4) | 55.6 (29.5-66.8) | 0.2 (0.0-6.0) |
| Grains & cereals | 11.9 (10.7-14.6) | 9.0 (5.3-15.8) | 75.0 (70.4-82.3) |
| Vegetables | 18.5 (12.1-33.5) | 12.9 (8.4-22.1) | 55.8 (40.6-70.8) |
| Fruits | 5.5 (3.4-8.1) | 3.5 (2.5-7.0) | 86.5 (78.3-95.5) |
| Dairy | 31.8 (25.5-39.5) | 34.4 (7.7-53.7) | 35.3 (11.7-55.3) |

Q, quartile.

## **Table S5a.** **AIC three mixture models for associations between macronutrient composition and energy intake from discretionary foods and the five food groups.**

| Model | Energy from discretionary foods | Energy from five food groups |
| --- | --- | --- |
| 1 (null model) | 10311 | 10374 |
| 2 (linear additive model) | 9877 | 9973 |
| 3 (quadratic model) | **9826** | **9919** |

AIC of models that were used to select the best-fitted model for assessing the association of macronutrient composition (% of energy) with energy proportion from discretionary foods and the five food groups. Model 1: null model (no dietary association); Model 2: linear additive associations (the ‘partition substitution model’ in nutritional epidemiology); and Model 3: quadratic (non-linear) associations. Models with minimal AIC scores are favored. When two models were within two AIC points of one another, the simplest model was selected. AIC Favoured in bold. Abbreviation: AIC, Akaike information criterion.

## **Table S5b. Coefficients for associations between macronutrient composition and energy intake from discretionary foods and the five food groups.**

|  | Estimate | SE | t value | p-value |
| --- | --- | --- | --- | --- |
| **Energy from discretionary foods (% of energy)** |  |  |  |  |
| *Model 3^1^*: *R^2^ = 38.5% ^2^, overall p<0.001* | |  |  |  |
| Protein | 41.33 | 45.50 | 0.91 | 0.36 |
| Carbohydrate | -17.62 | 15.92 | -1.11 | 0.27 |
| Fat | -29.22 | 20.67 | -1.41 | 0.16 |
| Protein: Carbohydrate | -254.33 | 80.69 | -3.15 | 0.002 |
| Protein: Fat | -364.72 | 62.00 | -5.88 | <0.001 |
| Carbohydrate: Fat | 132.64 | 25.92 | 5.12 | <0.001 |
| **Energy from the five food groups (% of energy)** |  |  |  |  |
| *Model 3: R^2^ = 35.1%, overall p<0.001* | | |  |  |
| Protein | -120.47 | 47.24 | -2.55 | 0.01 |
| Carbohydrate | 0.53 | 16.53 | 0.03 | 0.97 |
| Fat | -11.67 | 21.45 | -0.54 | 0.59 |
| Protein: Fat | 417.70 | 83.76 | 4.99 | <0.001 |
| Protein: Carbohydrate | 441.67 | 64.36 | 6.86 | <0.001 |
| Carbohydrate: Fat | -99.91 | 26.91 | -3.71 | <0.001 |

^1^ Model 3: quadratic (non-linear) model.

^2^ R^2^: Adjusted R-squared.

All models were adjusted for age, sex, initial weight loss, fiber intake, energy expenditure, BMI at baseline, fat-free mass at baseline, education, frequency of alcohol consumption, trial arm, and country.

Abbreviation: AIC, Akaike information criterion; Standard Error (SE).

## **Table S6. Macronutrient composition and energy intake across quintiles of discretionary food consumption.**

|  | Discretionary food consumption (% of energy) | | | | |
| --- | --- | --- | --- | --- | --- |
|  | Q1 (<15.2) | Q2 (15.2 - <24.0) | Q3 (24.0 - <33.1) | Q4 (33.1 - <43.8) | Q5 (≥43.8) |
| **Macronutrient composition (% of energy)** | |  |  |  |  |
| Protein | 24.1 (20.5-28.3) | 21.6 (18.6-25.1) | 20.0 (17.9-23.3) | 18.2 (16.2-21.0) | 15.9 (13.6-18.5) |
| Fat | 31.2 (26.0-37.9) | 32.8 (27.3-39.3) | 32.4 (28.3-38.2) | 32.8 (28.8-38.5) | 34.5 (30.1-39.6) |
| Carbohydrate | 42.3 (34.0-49.0) | 42.8 (36.1-48.7) | 43.8 (36.4-51.2) | 44.2 (36.8-50.3) | 45.7 (38.5-50.9) |
| Protein/non-protein ratio | 32.0 (26.2-40.4) | 28.4 (23.7-34.6) | 26.4 (22.1-32.3) | 23.7 (20.1-28.5) | 19.7 (16.6-24.2) |
| **Absolute intake (MJ)** |  |  |  |  |  |
| Protein | 1.6 (1.2-2.1) | 1.4 (1.1-1.8) | 1.4 (1.1-1.8) | 1.3 (1.1-1.7) | 1.2 (1.0-1.5) |
| Fat | 2.0 (1.5-3.0) | 2.2 (1.6-3.0) | 2.1 (1.7-3.1) | 2.4 (1.8-3.2) | 2.7 (2.1-3.3) |
| Carbohydrate | 2.7 (2.0-3.5) | 2.8 (2.0-3.6) | 3.0 (2.2-3.9) | 3.1 (2.4-4.1) | 3.6 (2.7-4.3) |
| Non-protein energy | 4.8 (3.7-6.2) | 5.0 (3.9-6.3) | 5.2 (4.2-6.7) | 5.5 (4.4-6.9) | 6.2 (4.8-7.5) |
| Total energy | 6.6 (5.0-8.4) | 6.5 (5.2-8.3) | 6.6 (5.7-8.6) | 7.1 (5.9-8.9) | 7.8 (6.2-9.3) |

Q, quintile.

## **Table S7. Generalized additive model outputs for food group consumption and total energy intake.**

|  | Parameter | edf | Ref. df | FS | P value | Corrected P value ^1^ |
| --- | --- | --- | --- | --- | --- | --- |
| **Total energy intake (MJ)** |  |  |  |  |  |  |
| **Discretionary** **foods** |  |  |  |  |  |  |
| s(discretionary foods, % of energy) | model a | 1.38 | 1.67 | 32.07 | <0.001 | <0.001 |
|  | model b | 2.60 | 3.30 | 42.71 | <0.001 | <0.001 |
|  | model c | 2.33 | 2.96 | 8.92 | <0.001 | <0.001 |
|  | model d | 2.58 | 3.27 | 2.05 | 0.09 | 0.09 |
| **The five food groups** |  |  |  |  |  |  |
| s(the five food groups, % of energy) | model a | 1.26 | 1.49 | 44.56 | <0.001 | <0.001 |
|  | model b | 2.29 | 2.91 | 44.87 | <0.001 | <0.001 |
|  | model c | 2.59 | 3.28 | 9.01 | <0.001 | <0.001 |
|  | model d | 2.53 | 3.20 | 1.97 | 0.11 | 0.11 |

^1^ P values corrected for 4 comparisons for each exposure using the Benjamini-Hochberg procedure.

Coefficients of generalized additive models for the association of energy proportions from discretionary foods and the five food groups with total energy intake.

Model a was an unadjusted model. Model b was adjusted for age, sex, initial weight loss, fiber intake, energy expenditure, BMI at baseline, fat-free mass at baseline, education, frequency of alcohol consumption, trial arm, and country. Model c was Model b plus saturated fatty acid intake, sodium intake, and sugar intake. Model d was Model c plus dietary protein content. Abbreviation: Edf, effective degrees of freedom; Ref.df, reference degrees of freedom; FS, F statistic.

## **Table S8. Comparison between participants retained in the complete-case analysis and the excluded participants.**

|  | **Participants in complete-case analysis** | **Excluded participants** | **p-value ^2^** |
| --- | --- | --- | --- |
| Participants | 1253 | 265 |  |
| Age, mean (SD), y | 44.8 (11.8) | 44.1 (12.4) | 0.40 |
| Fiber intake, mean (SD), g | 9 (8.4) | 9.3 (9.4) | 0.67 |
| Fat free mass, mean (SD), kg | 52.1 (10.1) | 51.9 (10) | 0.80 |
| Initial weight loss, mean (SD), kg | -11.7 (6.5) | -11.6 (6.6) | 0.91 |
| Sex, No. (%) |  |  | 0.03 |
| Male | 363 (29.0) | 95 (35.9) |  |
| Female | 890 (71.0) | 170 (64.1) |  |
| BMI, No. (%) ^1^ |  |  | 0.06 |
| <25 kg/m^2^ | 217 (17.4) | 61 (23.4) |  |
| 25- <30 kg/m^2^ | 533 (42.5) | 100 (38.5) |  |
| ≥30 kg/m^2^ | 503 (40.1) | 99 (38.1) |  |
| Country, No. (%) |  |  | <0.001 |
| UK (Leeds) | 445 (35.5) | 90 (34.0) |  |
| Denmark (Copenhagen), | 447 (35.7) | 53 (20.0) |  |
| Portugal (Lisbon) | 361 (28.8) | 122 (46.0) |  |
| Education, No. (%) |  |  | 0.05 |
| Low | 116 (9.3) | 20 (8.7) |  |
| Medium | 250 (20) | 64 (27.7) |  |
| High | 883 (70.7) | 147 (63.6) |  |
| Frequency of alcohol consumption, No. (%) |  |  | 0.06 |
| Every day | 27 (2.2) | 7 (3.0) |  |
| 5-6 times a week | 47 (3.8) | 3 (1.2) |  |
| 3-4 times a week | 132 (10.5) | 19 (8.0) |  |
| Twice a week | 190 (15.1) | 35 (14.8) |  |
| Once a week | 130 (10.4) | 37 (15.6) |  |
| <once a week | 727 (58.0) | 136 (57.4) |  |
| Trial arm, No. (%) |  |  | 0.62 |
| Control | 307 (24.5) | 68 (25.6) |  |
| Self-regulation and motivation | 313 (25.0) | 73 (27.6) |  |
| Stress and emotion regulation | 310 (24.7) | 65 (24.5) |  |
| Stress and emotion regulation + self-regulation and motivation | 323 (25.8) | 59 (22.3) |  |

^1^ Calculated as weight in kilograms divided by height in meters squared.

^2^ P values evaluated by ANOVA for continuous variables and χ2 test for categorical variables.

Abbreviation: BMI, body mass index; SD, standard deviation.

# **SUPPLEMENTAL FIGURES**


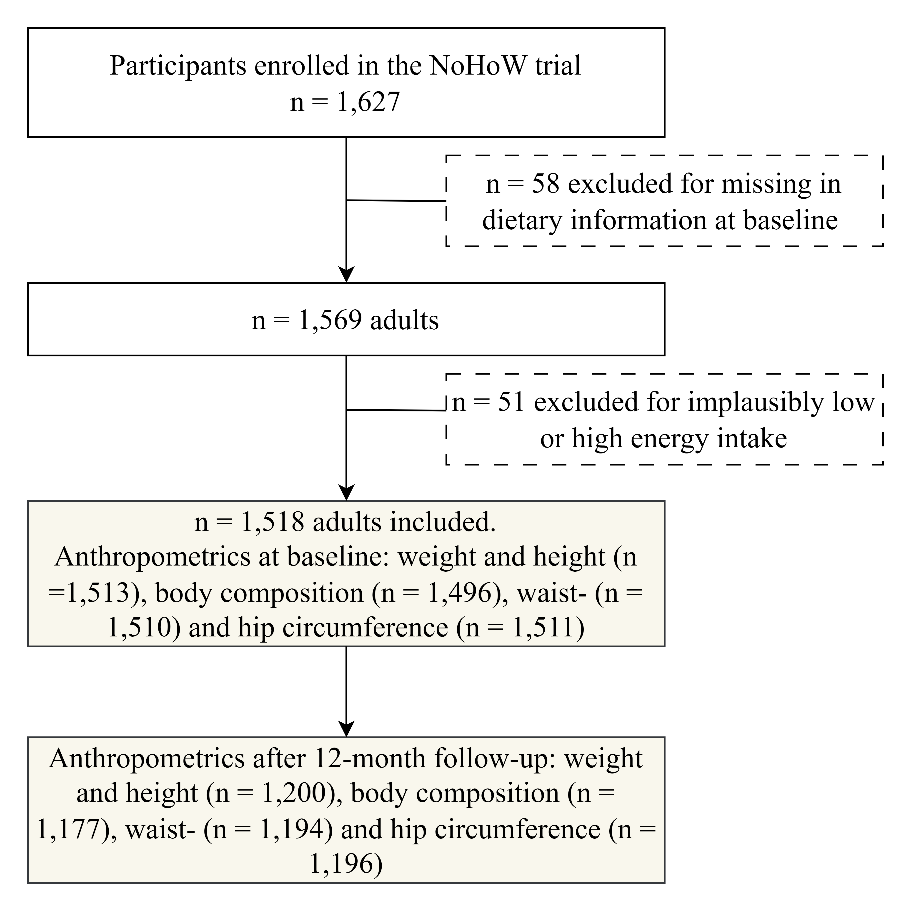


## **Figure S1. Flowchart of the study population.**


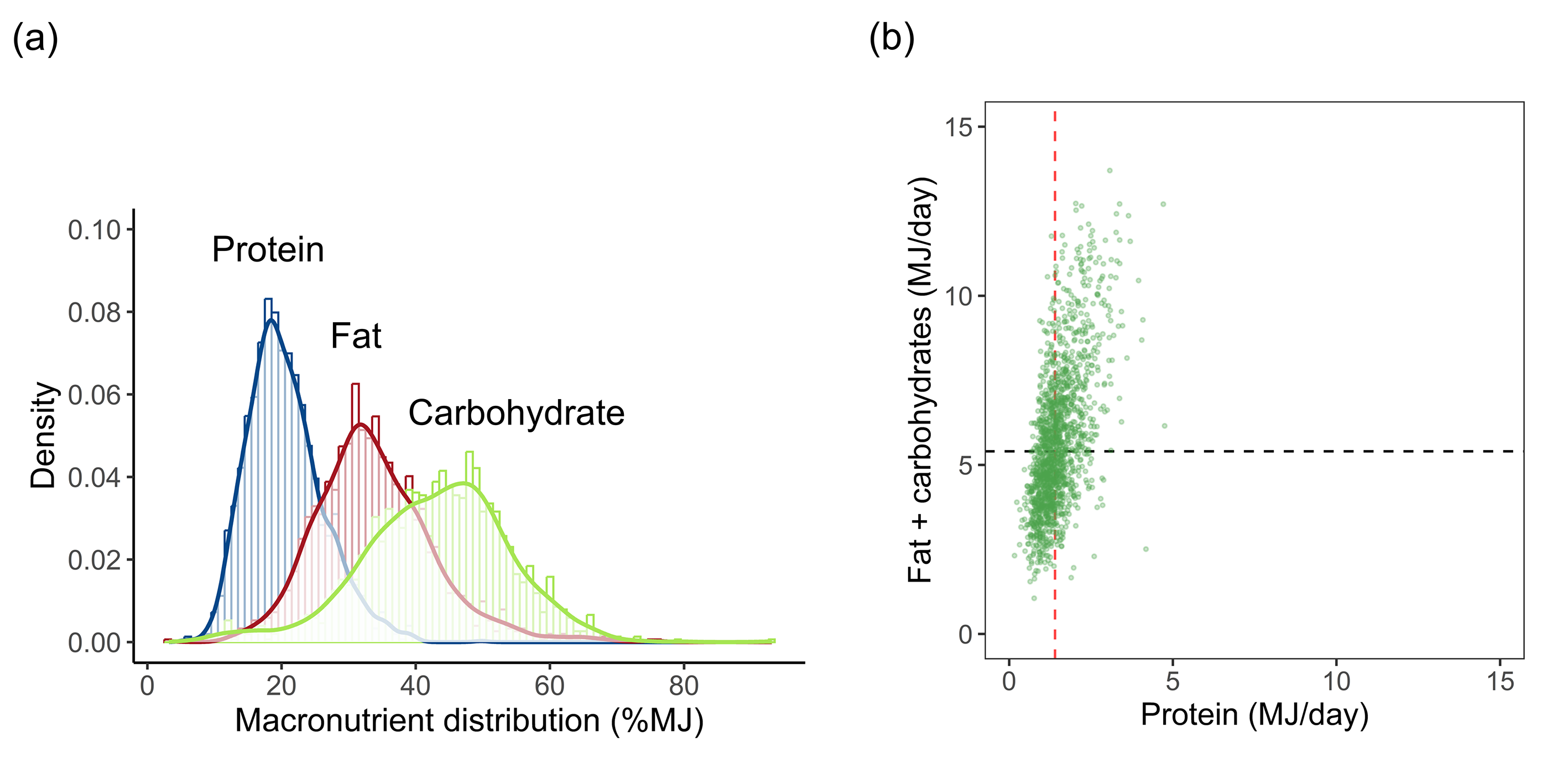


## **Figure S2. Variability of macronutrient intake.**

(a) Distribution of macronutrient intake. The protein distribution had a narrower and steeper peak and smaller tails than the fat and carbohydrate distributions. (b) Plot of protein energy versus non-protein energy intakes. Each point in the plot represents the absolute intake level of protein and non-protein energy in each participant. The red vertical line (crossing the median intake of protein energy) represents complete protein prioritization, in which absolute protein energy intake remains constant. The dark horizontal line (crossing the median intake of non-protein energy) represents complete non-protein prioritization, in which non-protein energy intake remains constant. The points approximated a vertical line with less variation on the protein axis than fat and carbohydrate energy.


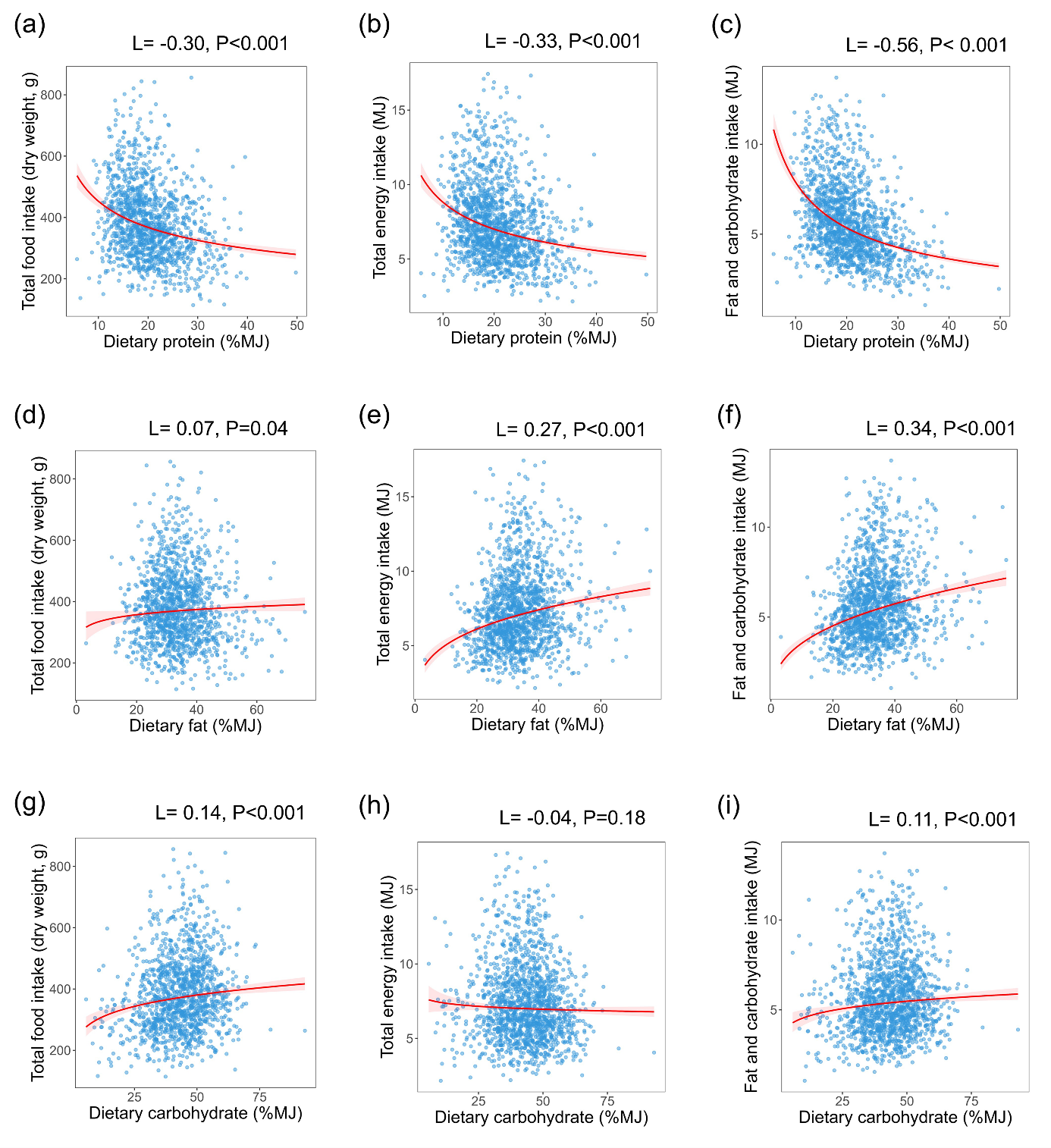


## **Figure S3.** **Association of proportional energy from each macronutrient with total food and energy intake.**

L indicates strength of leverage for each macronutrient (-1 signifies complete leverage, 0 means no leverage), derived from the log-log regression analysis (power functions). All models were adjusted for age, sex, initial weight loss, fiber intake, energy expenditure, BMI at baseline, fat-free mass at baseline, education, frequency of alcohol consumption, trial arm, and country. Statistics are given in S3 Table in Supplement. Abbreviation: L, leverage; MJ, megajoule.


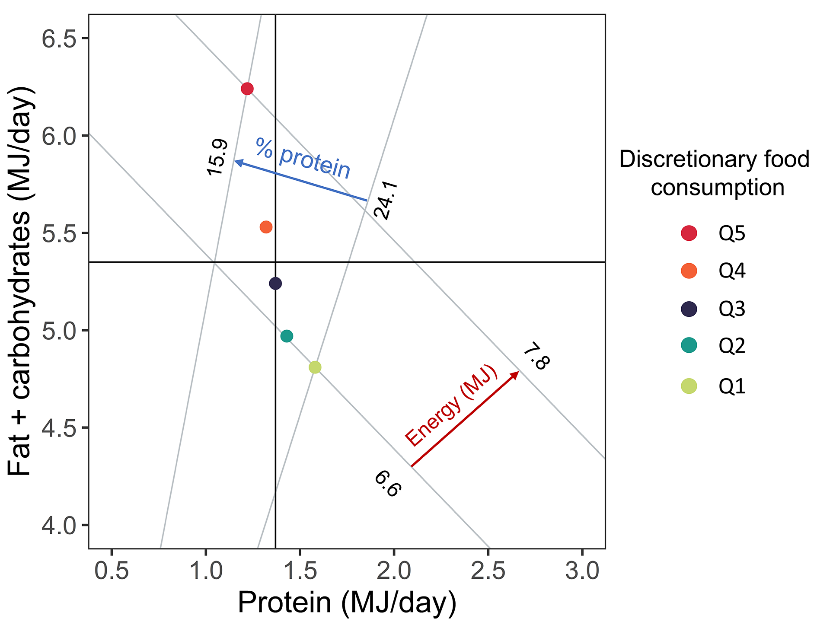


## **Figure S4. Protein and non-protein energy intake by quintile groups of discretionary food consumption.**

The grey radials from the origin represent diets with different protein-to-non-protein energy ratios (X/Y). The grey negatively sloped diagonals represent total energy intakes (X+Y). The five coloured points represent the median protein and non-protein energy in discretionary food consumption quintile groups. The dark vertical line (crossing the median intake of protein energy) represents complete protein prioritization. The dark horizontal line (crossing the median intake of non-protein energy) represents complete non-protein prioritization. Across quintiles of increasing discretionary food consumption, energy proportion from protein decreased; absolute intakes of fat and carbohydrate and total energy increased, while absolute protein intake remained relatively stable. Description values are given in Supplemental Table 6 in Supplement.


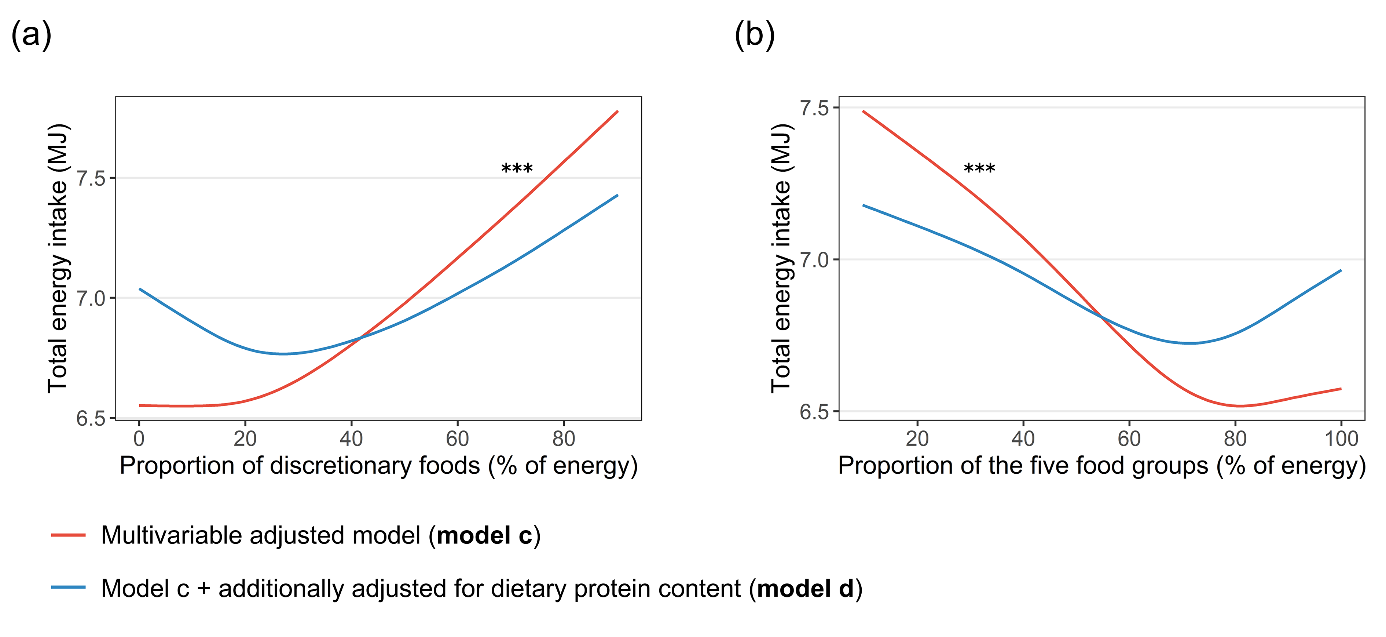


## **Figure S5.** **Association of proportion of discretionary foods and the five food groups with total energy intake.**

Smooth lines estimated by generalized additive models describe the association of energy proportions from discretionary foods and the five food groups with total energy intake. Model c was adjusted for age, sex, initial weight loss, fiber intake, energy expenditure, BMI at baseline, fat-free mass at baseline, education, frequency of alcohol consumption, trial arm, country, saturated fatty acid intake, sodium intake, and sugar intake. Model d was model c additionally adjusted for dietary protein content. Statistics are given in S7 Table in Supplement. ^***^ overall p-value < 0.001. Abbreviation: BMI, body mass index; MJ, megajoule.


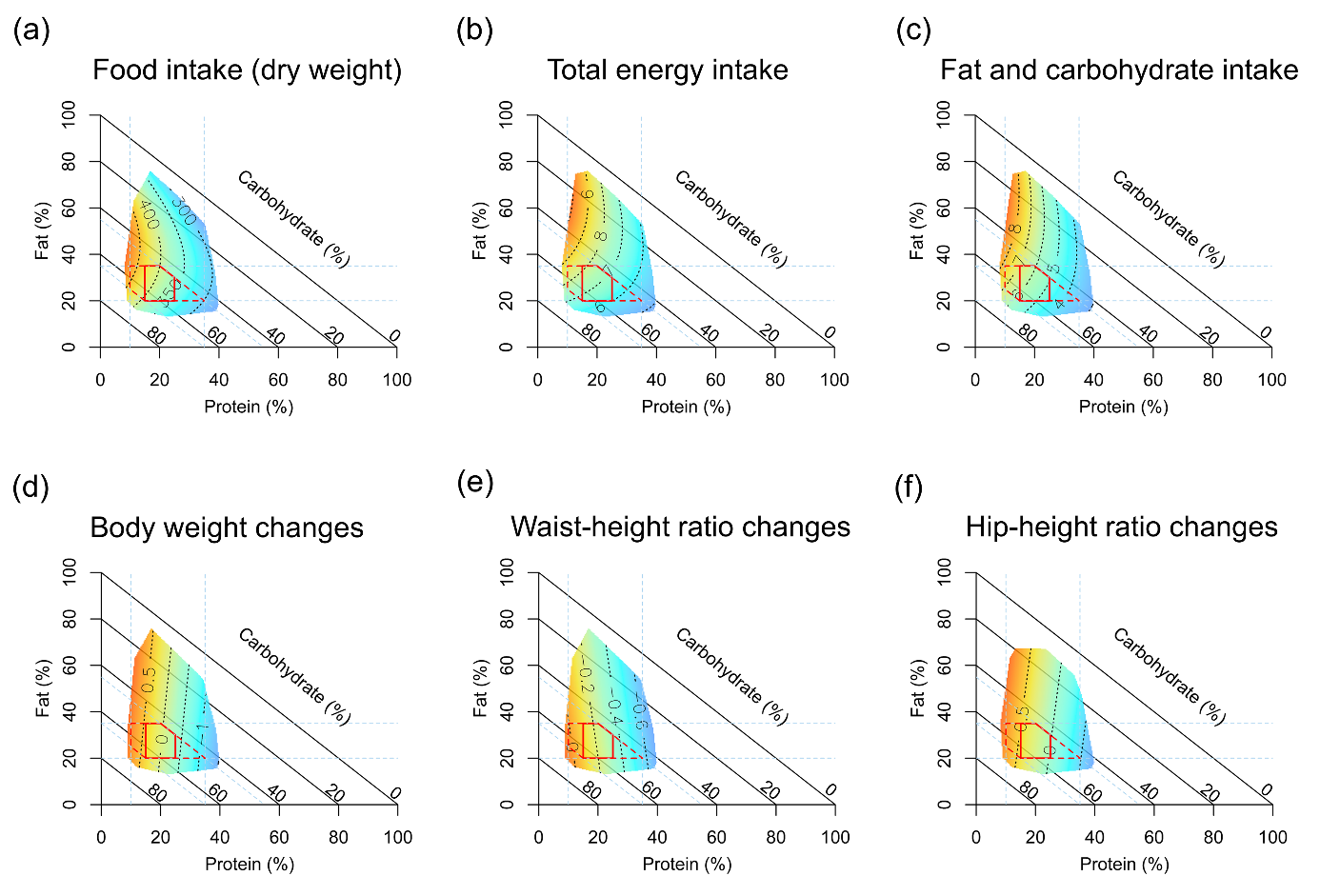


## **Figure S6.** **Association of dietary macronutrient composition with food and energy intake and 12-month weight loss maintenance after excluding participants with dietary information <4 days.**

Right-angled mixture triangle (RMT) plots show the association between energy proportions from protein, fat, and carbohydrate (%) and predicted (a) total food intake (dry weight, g), (b) total energy intake (MJ), (c) fat and carbohydrate intake (MJ), and 12-month changes in (d) body weight, (e) waist-, and (f) hip-height ratio after excluding participants with dietary information <4 days (remaining 1,194 participants). In the RMT, proportional energy from protein (%P), fat (%F), and carbohydrate (%C) sum to 100%. While %P and %F increase along their respective axes, %C increases across the diagonal lines with decreasing distance from the origin. On the plots, red represents the highest, while blue represents the lowest value of the outcomes. Mixture models estimated colored surfaces. Models were adjusted for age, sex, initial weight loss, fiber intake, energy expenditure, BMI at baseline, fat-free mass at baseline, education, frequency of alcohol consumption, trial arm, and country. For reference, the red solid polygon represents the Acceptable Macronutrient Distribution Range (AMDR) for Australia and New Zealand (%P=15–25, %F=20–35, %C=45–65), and the red dotted polygon in the background shows the AMDR for the USA (%P=10–35, %F=20–35, %C=45–65). Abbreviation: AMDR, Acceptable Macronutrient Distribution Range; MJ, megajoule; RMT, Right-angled mixture triangle.


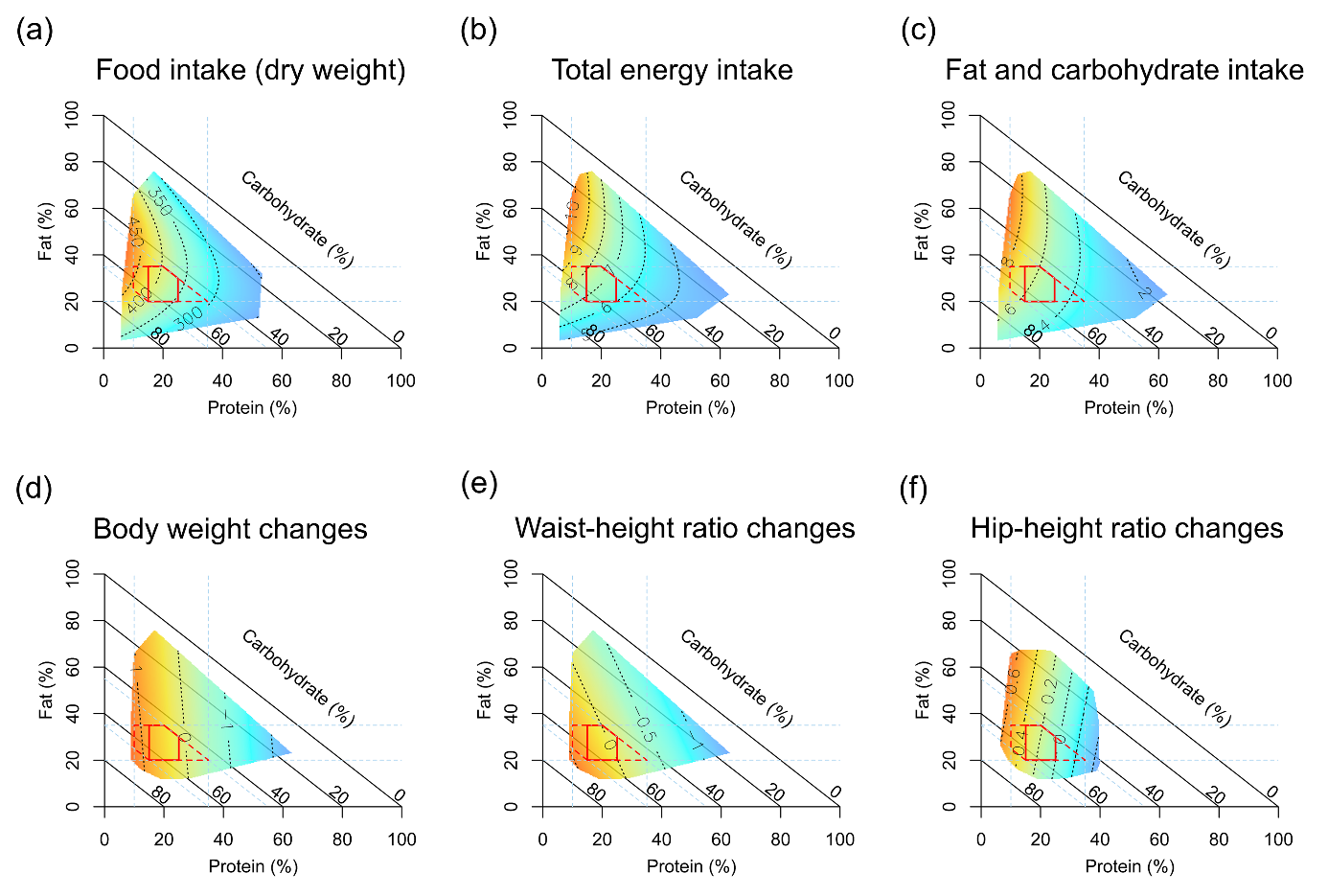


## **Figure S7. Association of dietary macronutrient composition with food and energy intake and 12-month weight loss maintenance after multiple imputations for missing covariates.**

Right-angled mixture triangle (RMT) plots show the association between energy proportions from protein, fat, and carbohydrate (%) and predicted (a) total food intake (dry weight, g), (b) total energy intake (MJ), (c) fat and carbohydrate intake (MJ), and 12-month changes in (d) body weight, (e) waist-, and (f) hip-height ratio after multiple imputation. In the RMT, proportional energy from protein (%P), fat (%F), and carbohydrate (%C) sum to 100%. While %P and %F increase along their respective axes, %C increases across the diagonal lines with decreasing distance from the origin. On the plots, red represents the highest, while blue represents the lowest value of the outcomes. Mixture models estimated colored surfaces. Models were adjusted for age, sex, initial weight loss, fiber intake, energy expenditure, BMI at baseline, fat-free mass at baseline, education, frequency of alcohol consumption, trial arm, and country. For reference, the red solid polygon represents the Acceptable Macronutrient Distribution Range (AMDR) for Australia and New Zealand (%P=15–25, %F=20–35, %C=45–65), and the red dotted polygon in the background shows the AMDR for the USA (%P=10–35, %F=20–35, %C=45–65). Abbreviation: AMDR, Acceptable Macronutrient Distribution Range; MJ, megajoule; RMT, Right-angled mixture triangle.


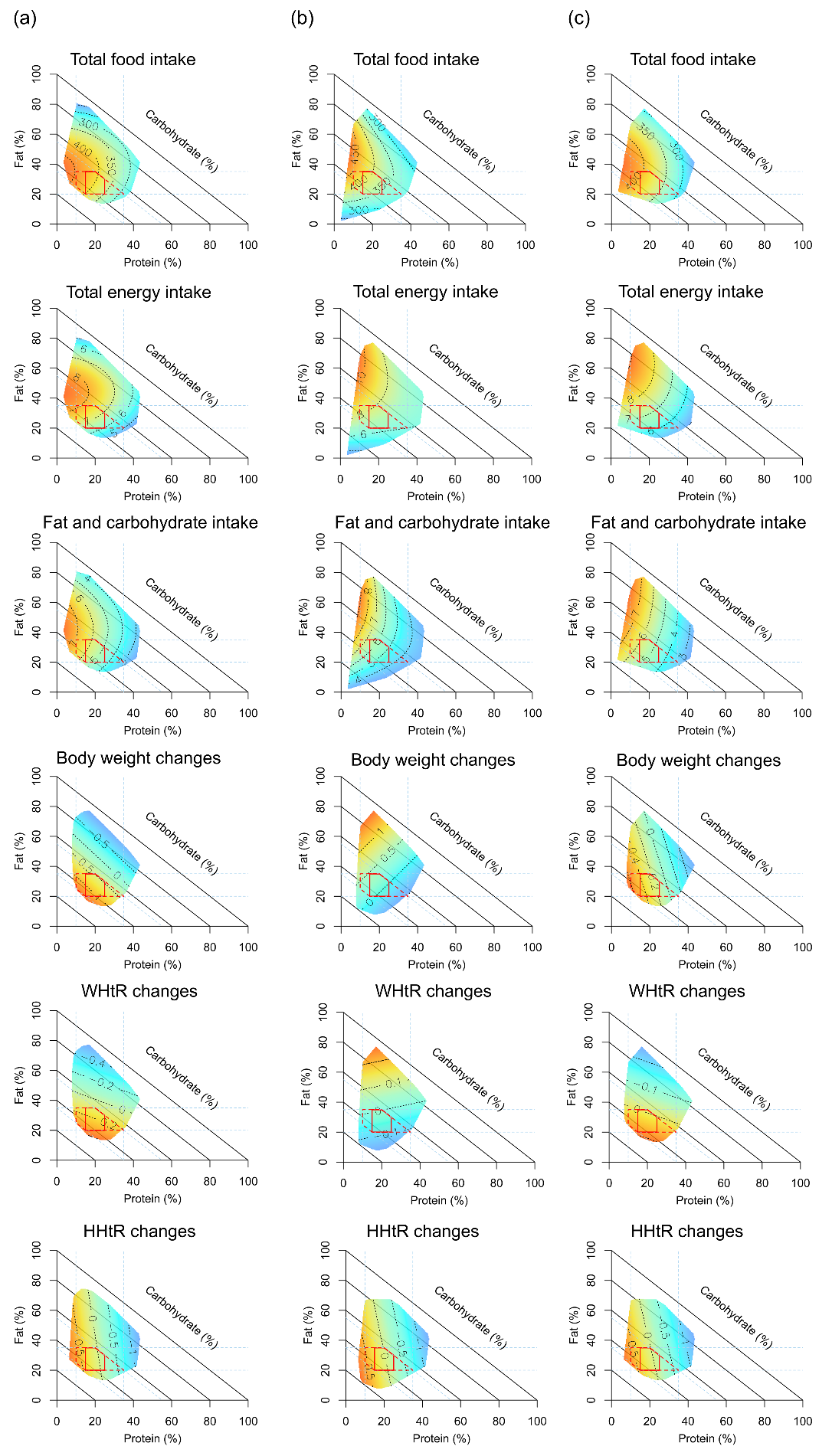


## **Figure S8. Association of dietary macronutrient composition with food and energy intake and 12-month weight loss maintenance after adjusting underreporting.**

Right-angled mixture triangle (RMT) plots show the association between energy proportions from protein, fat, and carbohydrate (%) and predicted total food intake (dry weight), total energy intake (MJ), fat and carbohydrate intake (MJ), 12-month changes in body weight, waist-height ratio, and hip-height ratio after adjusting potential systematic underreporting of fat and carbohydrate. We assumed that underreporting energy intake in this study was caused by either (a) 100% dietary fat, (b) 100% dietary carbohydrate, or (c) 50% dietary fat and 50% carbohydrate. All adjustments for fat and carbohydrate intake were conducted among those participants with obesity at baseline (BMI ≥30 kg/m^2^). In the RMT, proportional energy from protein (%P), fat (%F), and carbohydrate (%C) sum to 100%. While %P and %F increase along their respective axes, %C increases across the diagonal lines with decreasing distance from the origin. On the plots, red represents the highest, while blue represents the lowest value of the outcomes. Mixture models estimated colored surfaces. Models were adjusted for age, sex, initial weight loss, fiber intake, energy expenditure, BMI at baseline, fat-free mass at baseline, education, frequency of alcohol consumption, trial arm, and country. For reference, the red solid polygon represents the Acceptable Macronutrient Distribution Range (AMDR) for Australia and New Zealand (%P=15–25, %F=20–35, %C=45–65), and the red dotted polygon in the background shows the AMDR for the USA (%P=10–35, %F=20–35, %C=45–65). Abbreviation: AMDR, Acceptable Macronutrient Distribution Range; HHtR, hip-height ratio; MJ, megajoule; RMT, Right-angled mixture triangle; WHtR, waist-height ratio.

# **SUPPLEMENTAL REFERENCES**

1. Council NHaMR. *Australian Dietary Guidelines*. 2013.

2. Lewis HD, Jr. Missing data in clinical trials. *N Engl J Med*. Dec 27 2012;367(26):2557; author reply 2557-8. doi:10.1056/NEJMc1213388

3. Sterne JA, White IR, Carlin JB, et al. Multiple imputation for missing data in epidemiological and clinical research: potential and pitfalls. *BMJ*. Jun 29 2009;338:b2393. doi:10.1136/bmj.b2393

4. Lissner L, Troiano RP, Midthune D, et al. OPEN about obesity: recovery biomarkers, dietary reporting errors and BMI. *Int J Obes (Lond)*. Jun 2007;31(6):956-61. doi:10.1038/sj.ijo.0803527

5. Heitmann BL, Lissner L, Osler M. Do we eat less fat, or just report so? *Int J Obes Relat Metab Disord*. Apr 2000;24(4):435-42. doi:10.1038/sj.ijo.0801176

6. Heitmann BL, Lissner L. Dietary underreporting by obese individuals--is it specific or non-specific? *BMJ*. Oct 14 1995;311(7011):986-9. doi:10.1136/bmj.311.7011.986
